# Development of a cloud-based flow rate tool for eNAMPT biomarker detection

**DOI:** 10.1093/pnasnexus/pgae173

**Published:** 2024-04-24

**Authors:** Bailey C Buchanan, Yisha Tang, Hannah Lopez, Nancy G Casanova, Joe G N Garcia, Jeong-Yeol Yoon

**Affiliations:** Department of Biomedical Engineering, The University of Arizona, 1127 E. James E. Rogers Way, Tucson, AZ 85721, USA; Department of Biomedical Engineering, The University of Arizona, 1127 E. James E. Rogers Way, Tucson, AZ 85721, USA; Department of Neuroscience, The University of Arizona, 1040 E. 4th Street, Tucson, AZ 85721, USA; Center for Inflammation Science and Systems Medicine, The Herbert Wertheim UF Scripps Research Institute for Biomedical Innovation and Technology, University of Florida, 120 Scripps Way, Jupiter, FL 33458, USA; Center for Inflammation Science and Systems Medicine, The Herbert Wertheim UF Scripps Research Institute for Biomedical Innovation and Technology, University of Florida, 120 Scripps Way, Jupiter, FL 33458, USA; Department of Biomedical Engineering, The University of Arizona, 1127 E. James E. Rogers Way, Tucson, AZ 85721, USA

**Keywords:** acute respiratory distress syndrome, paper microfluidics, smartphone, capillary action, Google Colab

## Abstract

Increased levels of extracellular nicotinamide phosphoribosyltransferase (eNAMPT) are increasingly recognized as a highly useful biomarker of inflammatory disease and disease severity. In preclinical animal studies, a monoclonal antibody that neutralizes eNAMPT has been generated to successfully reduce the extent of inflammatory cascade activation. Thus, the rapid detection of eNAMPT concentration in plasma samples at the point of care (POC) would be of great utility in assessing the benefit of administering an anti-eNAMPT therapeutic. To determine the feasibility of this POC test, we conducted a particle immunoagglutination assay on a paper microfluidic platform and quantified its extent with a flow rate measurement in less than 1 min. A smartphone and cloud-based Google Colab were used to analyze the flow rates automatically. A horizontal flow model and an immunoagglutination binding model were evaluated to optimize the detection time, sample dilution, and particle concentration. This assay successfully detected eNAMPT in both human whole blood and plasma samples (diluted to 10 and 1%), with the limit of detection of 1–20 pg/mL (equivalent to 0.1–0.2 ng/mL in undiluted blood and plasma) and a linear range of 5–40 pg/mL. Furthermore, the smartphone POC assay distinguished clinical samples with low, mid, and high eNAMPT concentrations. Together, these results indicate this POC assay, which utilizes low-cost materials, time-effective methods, and a straightforward immunoassay (without surface immobilization), may reliably allow rapid determination of eNAMPT blood/plasma levels to advantage patient stratification in clinical trials and guide ALT-100 mAb therapeutic decision-making.

**Significance Statement**

Extracellular nicotinamide phosphoribosyltransferase (eNAMPT) is a major damage-associated molecular pattern protein and a viable therapeutic target in dysregulated innate immunity-mediated inflammation. For example, ALT-100 mAb is a humanized eNAMPT-neutralizing monoclonal antibody currently in clinical trials for patients with inflammatory lung injury. The rapid detection of eNAMPT concentration in plasma samples at the point of care (POC) would be of high utility in determining the administration of anti-eNAMPT therapeutic. Currently available eNAMPT assays are not rapid and labor-intensive; however, a POC test that reliably measures plasma eNAMPT levels would advantage patient stratification. We now report the development of a low-cost, cloud-based flow rate POC test on paper clips that achieves a clinically useful limit of detection to guide ALT-100 mAb therapeutic decision-making.

## Introduction

Acute respiratory distress syndrome (ARDS) is a severe inflammatory respiratory illness caused by sepsis, trauma, pneumonia, and ventilator-induced lung injury and has a high mortality rate (1, 2). Extracellular nicotinamide phosphoribosyltransferase (eNAMPT) plays a role in the biosynthesis of nicotinamide adenine dinucleotide (NAD) and apoptosis pathways (3, 4). In addition, eNAMPT is upregulated in many inflammatory disorders (4). Moreover, eNAMPT has been discovered as a biomarker for ARDS, with increasing eNAMPT concentrations leading to more severe ARDS cases (1, 5, 6). When ARDS stimuli such as hypoxia, ventilator stress, and infection are present, eNAMPT expression is induced (7–9). Following this, eNAMPT activates toll-like receptor 4 (TLR4), which then has profound effects on the NFκB-driven inflammatory pathway (1, 5, 10). Preclinical studies confirmed the utility of this anti-eNAMPT, a neutralizing monoclonal antibody (mAb), in reducing the magnitude of the inflammatory response driven by eNAMPT/TLR4 signaling (1, 10–12). This anti-eNAMPT could reduce the severity and mortality experienced in patients with ARDS.

Bermudez et al. conducted a preclinical study in Sprague Dawley rats and Yucatan minipigs, confirming the efficacy of neutralizing anti-eNAMPT ALT-100 mAb. It significantly reduced the severity of lung injury in their animal models (1). Therefore, it is hypothesized that administering the anti-eNAMPT ALT-100 mAb could successfully treat human patients with ARDS (1, 10, 11). Currently, no U.S. Food & Drug Administration-approved therapies exist that can aid in reducing the severity of lung injuries in ARDS patients (1, 13). To increase the chance of a successful clinical trial, we should be able to identify which patients have high levels of eNAMPT, as ARDS patients with higher levels of eNAMPT are hypothesized to respond the best to anti-eNAMPT ALT-100 mAb (10, 14). In addition, it is also essential to measure eNAMPT levels quickly and cost effectively to monitor the efficacy of the mAb drug therapy. Finally, monitoring eNAMPT levels is also crucial following the potential implementation of the anti-eNAMPT ALT-100 mAb as a drug therapy for ARDS patients.

A point-of-care (POC) device that measures eNAMPT levels would be crucial for patients and physicians in determining when to administer the mAb drug and its dose. A POC test would allow the patient or physician to determine whether (i) eNAMPT levels are low and no drug administration is needed or (ii) eNAMPT levels are high and drug administration is required. Since anti-eNAMPT is a neutralizing mAb, administering excess amounts could lead to adverse downstream effects (15, 16). Anti-eNAMPT ALT-100 mAb should not be administered when the eNAMPT level is normal since it plays a role in NAD biosynthesis and apoptosis pathways. Due to this, it is essential only to administer the ALT-100 mAb drug when eNAMPT levels are high, indicating the need for the neutralizing mAb drug.

**Fig. 1.**
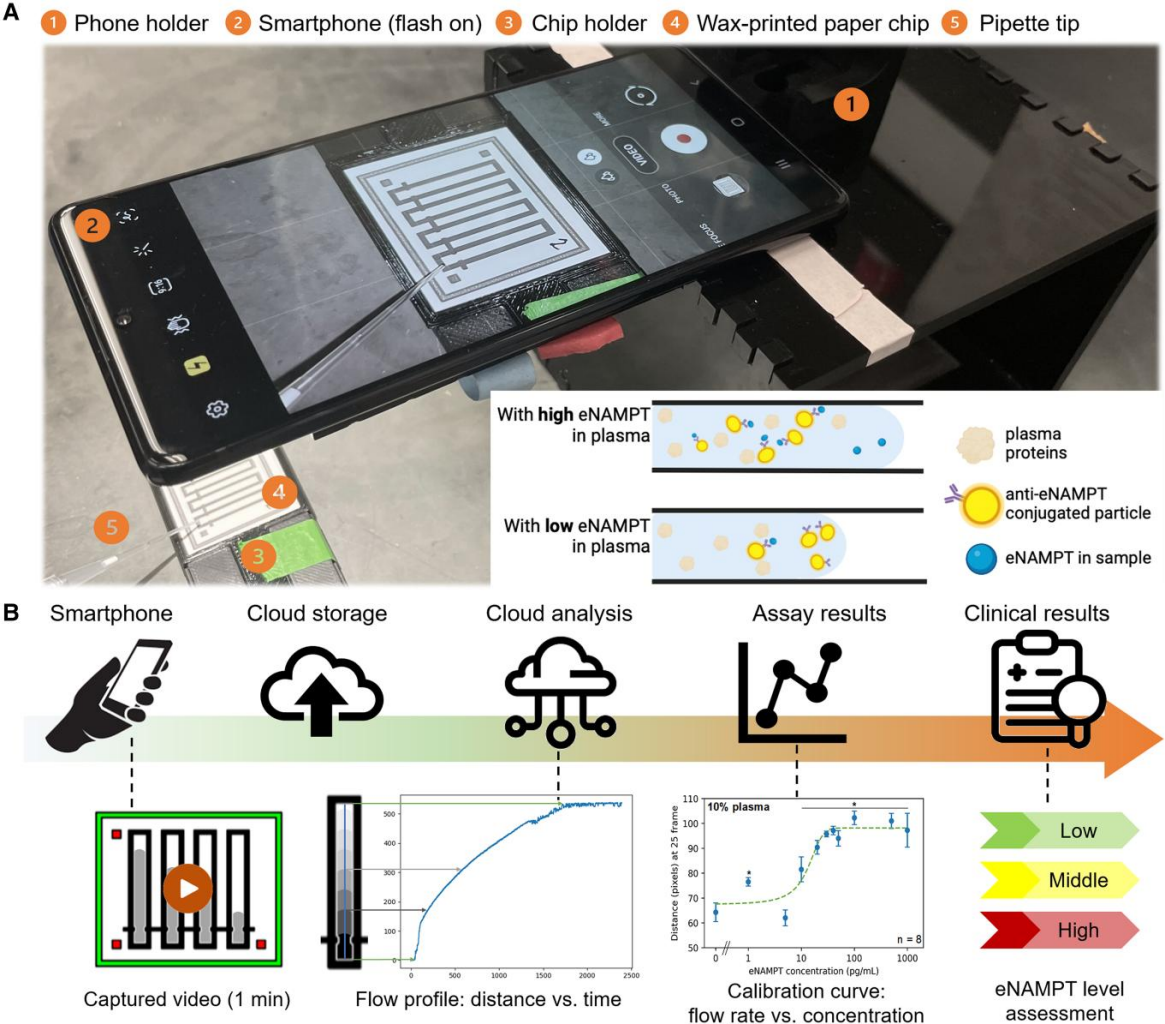
Systematic diagram of the cloud-based flow rate tool for eNAMPT detection. A) The assay included a smartphone on the phone holder, a chip holder for a wax-printed paper chip, and a pipette loading the sample to the channel inlet. A smartphone captures a video while the sample flows through the channel. The eNAMPT in samples can induce the preloaded antibody-conjugated particles to agglutinate, making it difficult to reach the liquid–gas interface (wetting front), resulting in different flow rates depending on the eNAMPT concentration, as shown in the inset. B) A scheme of cloud-based data processing. The video of the flow captured by the smartphone is stored and processed in the cloud. Flow rate profiles were then extracted and analyzed using developed Python scripts running on the cloud. A calibration curve for the assay is obtained with the flow distances at an optimized time point as the signal with varying eNAMPT concentration. The assay can qualitatively assess low, medium, and high eNAMPT levels in clinical plasma samples based on the calibration curve.

This work demonstrates a paper microfluidic platform toward a time- and cost-effective POC test for eNAMPT. Anti-eNAMPT ALT-100 mAb is covalently conjugated to the submicron particles and then preloaded into the beginning portion of the microfluidic channel of the paper chip, as depicted in Fig. 1. (While fluorescent particles were used to image the particles on paper chips to confirm their distribution, as shown in the Fig. S1, fluorescence is unnecessary for this assay as the detection is purely based on the flow rate.) Following this, samples with various concentrations of eNAMPT are loaded into each microfluidic channel. The binding between the anti-eNAMPT ALT-100 mAb and the target eNAMPT triggers the immunoagglutination of submicron particles. As a result, agglutinated particles are more likely to be left behind during the capillary action through the paper microfluidic channel. In contrast, nonaggregated particles can adsorb on the “wetting front,” i.e. the liquid–gas interface within the paper fibers. Such a difference leads to changes in interfacial tension and capillary flow rate. A 1-min video is captured to obtain the flow rate and used to identify and quantify the eNAMPT antigens in the sample.

Previously, we have demonstrated a similar method for detecting neutralizing antibodies against SARS-CoV-2 from human saliva samples (17), where competitive particle immunoassay was used. Without target presence in the sample, the receptor-coated particles were captured by the target molecules immobilized on a paper substrate, leading to a faster capillary flow. With target presence, the receptor-coated particles preferred to bind to the free targets than the immobilized target, flowing through the capillary action, leading to a slower flow rate. In this work, we demonstrate a noncompetitive particle immunoagglutination assay. Compared to competitive immunoassays, it offers a higher sensitivity, specificity, and a broader linear range (18–21). In addition, it does not require the preimmobilization of target molecules. However, the distinction between immunoagglutination and nonimmunoagglutiation has not been demonstrated with the capillary flow rates.

The working principle behind the capillary flow rate detection is depicted in the inset of Fig. 1A. With fewer particles in the wetting front (resulting from particle immunoagglutination), the interfacial tension is close to the maximum, and the capillary flow rate is also high, following the Lucas–Washburn (L-W) law (22). Conversely, with a lower concentration of eNAMPT present, less immunoagglutination occurs, leading to more antibody-conjugated particles in the wetting front, which would decrease the capillary flow rate. Thus, the flow rate is positively correlated to the eNAMPT concentration. Such particle immunoagglutination should alter the liquid's interfacial tension and/or dynamic viscosity, leading to the deviation from the L-W law. Therefore, optimizing the time for flow rate determination is essential, preferably when such deviation is maximum. In this work, we attempted to optimize such detection time through a series of horizontal flow modeling and immunoagglutination binding modeling, which have not been demonstrated previously.

While numerous paper microfluidic assays coupled with smartphone detection methods have been demonstrated, including our previous works (23–25), they have predominantly utilized colorimetry, fluorescence, or light scattering, which require rather complicated optical detection settings, e.g. a need for a dark chamber, a compensation algorithm for ambient lighting variations, or a separate light source, etc.

This work demonstrates a quick, low-cost, and easy-to-use solution for detecting eNAMPT from human blood and plasma samples (including clinical samples). It requires only a paper microfluidic chip (preloaded with anti-eNAMPT conjugated polystyrene particles) and a smartphone's video capture feature (Fig. 1). It monitors the capillary flow rates in less than 1 min, which is not affected by ambient lighting variations and requires neither a separate light source nor optical filters. It also demonstrates a cloud-based monitoring system that can analyze and show the results on a smartphone while the analysis is conducted remotely on a cloud server.

## Materials and methods

### Reagents and samples

eNAMPT antigen and ALT-100 mAb (anti-eNAMPT monoclonal antibody [mAb]) were obtained from Aqualung Therapeutics (Juno Beach, FL, USA). Details on the generation of ALT-100 have been previously reported (1). eNAMPT antigen was spiked into 10 and 1% pooled whole human blood (BioIVT, Westbury, New York, USA) at final concentrations of 0, 1, 5, 10, 20, 30, 40, 50, 100, 500, and 1,000 pg/mL. The same whole human blood was also spun down at 2,000 *g* for 10 minutes to obtain plasma. They were diluted to 10 and 1% and spiked with eNAMPT antigen to obtain final concentrations of 0, 1, 5, 10, 20, 30, 40, 50, 100, 500, and 1,000 pg/mL.

Clinical data and plasma samples were obtained from fifteen subjects enrolled in the Acute Respiratory Distress Network study (accession number HLB00500606a). Informed consents were obtained from all participants. Of the fifteen clinical samples, five of them fell into the low eNAMPT concentration range, as determined by enzyme-linked immunosorbent assay (ELISA), from 8.6–11.9 ng/mL, five fell into the middle eNAMPT concentration range from 18.0–23.1 ng/mL, and the remaining five fell into the high eNAMPT concentration range from 86.8–197 ng/mL (26). This study was approved by the University of Arizona's institutional review board (IRB 1312168664R001). Plasma was diluted to 10%, 1%, 0.1%, and 0.01%. The eNAMPT concentrations were separately identified with ELISA, as described in the previous work (26). Subject characteristics are presented in Table S1.

### Paper chip fabrication

Unisart® nitrocellulose membrane CN95 (capillary speed is 65–115 s/40 mm; 240–270 μm thickness; pore size is approximately 15 μm; Sartorius, Goettingen, Germany) was used as a paper substrate. The channels were printed by a wax printer (ColorQube 8550; Xerox, Norwalk, CT, USA). The chip had a design of four rectangular channels of 2.1 cm × 0.3 cm with a loading area of 0.3 cm × 0.3 cm at the beginning of each channel (Fig. 1B). The chips were placed face up on a hot plate set to 120°C for 2 minutes. A metal weight was placed on top to apply even pressure throughout the entire chip and to allow the wax to melt evenly through the depth of the paper. The chips were allowed to cool and placed in a refrigerator in a sealed container or bag until use.

### Conjugation of anti-eNAMPT to the particles

Anti-eNAMPT ALT-100 mAb was covalently conjugated via carbodiimide chemistry to yellow–green fluorescent, carboxylated polystyrene particles (diameter is 0.5 μm; Magsphere Inc., Pasadena, CA, USA) following the protocol available at protocols.io: https://doi.org/10.17504/protocols.io.bhsvj6e6 The final concentration of the anti-eNAMPT conjugated fluorescent particles was 0.0318 µg/µL after the conjugation.

### Flow rate assay procedure

Before the assays, 1 µL of the anti-eNAMPT conjugated particles at a concentration of 0.0318 µg/µL was loaded onto the loading area of each channel. The antibody-particle suspension flowed through the channel via capillary action and dried for five minutes. The antibody-conjugated particles were distributed within the paper fibers of each channel. The particle-loaded paper chips were mass-produced and stored in a refrigerator for future use.

The prepared chip was placed into a chip holder that was 3D-printed with the Creality Ender 3 (Creality, Shenzhen, China) using PETG filament (Overture, Wilmington, DE, USA). A smartphone (Samsung Galaxy S10 Lite; Samsung Electronics America, Inc., NJ, USA) was placed on the phone holder, as shown in Fig. 1A. 4 µL of a sample (diluted whole blood or plasma containing eNAMPT) was loaded onto each channel, and a video was captured for 1 min until the sample flowed to the end of a channel. The default camera application was used with the flash on. All eNAMPT-spiked or clinical samples were assayed on two chips, giving eight measurements. The clinical plasma samples were diluted to 0.01% and assayed using the lower concentration (0.0159 µg/µL) of anti-eNAMPT conjugated particles.

### Flow rate data analysis

Video data analysis was executed in the cloud environment (Fig. 1B). Video files capturing flow dynamics were synchronized to Google Drive for storage and imported into Google Colaboratory (Colab) for computational processing. The OpenCV package and the custom Python script were utilized to extract the flow profile over time, i.e. the wetting front along the central axis of each channel frame by frame (Code S1). Following this, the code obtained a flow profile delineating flow distance versus frame number for each channel as the sample flowed through the channel. These flow profiles were automatically saved in Google Sheets. The capillary flow was then modeled using the L-W equation, which inferred an optimum time point providing maximal antigen concentration separation. Employing the flow distance at the optimized time point as the signal, a dose-responsive calibration curve for this immunoagglutination-induced flow assay was established and validated with clinical samples afterward. The calibration curve of the immunoassay at each dilution was fitted individually with the sigmoid curve.

### Horizontal capillary flow modeling

The horizontal capillary flow in porous media can be described by the L-W law, in which the distance of liquid movement *L* and the time *t* satisfy a diffusion relationship as $L=\sqrt{Dt}=\sqrt{\frac{R\gamma_{LG}\cos\;\theta }{2\mu }t}$ (27, 28). Here, *D* is the diffusion coefficient depending on the capillary tube size (replaced with the capillary radius *R* in this work) and liquid properties such as interfacial tension *γ*_LG_ at the liquid–gas interface, contact angle *θ*, and dynamic viscosity *μ*. Two methods were used to estimate the diffusion coefficient and investigate its difference among various antigen concentrations. One is that the diffusion coefficient *D* was computed at every time frame, from *L*^2^/*t* in the flow rate plot. The time-serial flow profile of each channel was smoothed by moving average and then averaged with other channels in one chip, representing one eNAMPT concentration. Another is fitting the entire flow profile (distance versus time) with the L-W equation to estimate the overall diffusion coefficient for each antigen concentration. Again, each channel's flow was fitted with the nonlinear square root formula to find likely parameters describing its own. Initially, fittings were made for the entire time frame (0 to 1,500 frames = 0 to 50 s), followed by shorter time frames, e.g. 0 to 500 frames, 0 to 200 frames, 0 to 100 frames, etc.

To elucidate the predominant factors influencing the capillary flow dynamics, interfacial tension and viscosity were measured using the pendant drop method and an Ostwald viscometer (part number 1831; Yucheng Tech, Zhejiang, China), respectively. In the pendant drop analysis, the antibody-conjugated particle was mixed with a range of eNAMPT concentrations at a fixed volume ratio of 1:4, and a pear-shaped pendant drop was formed under a blunt-end needle. Pictures of the drop were taken and analyzed using the Pendant Drop plugin within ImageJ (U.S. National Institutes of Health, Bethesda, MD, USA). For viscosity measurements, due to the limited volume of antibody-conjugated particles, the mixture of particles and eNAMPT solutions was further diluted by a factor of 50 to increase the volume to a suitable level. This diluted mixture was loaded into the Ostwald viscometer, and the time interval for the liquid level to descend from one marker to another was recorded. The relative viscosity was normalized with the deionized (DI) water results measured on the same day.

### Immunoagglutination modeling

A mathematical model of immunoagglutination was adapted from (29, 30) to simulate how agglutination varies with different initial amounts of antigen and antibody-conjugated particles introduced into the system. In this assay, antigens encounter particles within the nitrocellulose paper. Since the fiber network may hinder their binding reaction, the immunoagglutination reaction is spatially uneven and insufficient. Therefore, we considered two types of binding reactions. (i) The primary binding of one antibody-conjugated particle (Abp) with one antigen (Ag), along with its dissociation: Abp + Ag ⇌ Abp·Ag, with the association and dissociation constants *k*_on,1_ and *k*_off,1_. (ii) The secondary binding event between the antigen-bound particles (Abp·Ag) with free particles (Abp): Abp + Abp·Ag ⇌ Abp2·Ag, with the constants *k*_on,2_ and *k*_off,2_, or that between the antigen-bound particles (Abp·Ag) with free antigens (Ag): Ag + Abp·Ag ⇌ Abp·Ag2, with the constant *k*_on,3_. Abp2·Ag represents particle immunoagglutination with two particles (Abp2), while Abp·Ag2 does not (with only one particle Abp), the model strives to solve for Abp2·Ag. Dissociation of Abp·Ag2 (and the constant *k*_off,3_) is not considered to simplify the model, since it does not make a difference to the particle immunoagglutination whether the particle has one or two antigens bound to it. The model is described by a series of differential equations as a function of time (*t*):


$$\begin{array}{rl} \frac{d[\mathrm{Abp}\cdot \mathrm{Ag}]}{dt} & =k_{\mathrm{on},1}[\mathrm{Abp}][\mathrm{Ag}]-k_{\mathrm{off},1}[\mathrm{Abp}\cdot \mathrm{Ag}]-k_{\mathrm{on},2}[\mathrm{Abp}][\mathrm{Abp}\cdot \mathrm{Ag}]\\ & +k_{\mathrm{off},2}[\mathrm{Abp}2\cdot \mathrm{Ag}]-k_{\mathrm{on},3}[\mathrm{Abp}\cdot \mathrm{Ag}][\mathrm{Ag}] \end{array}$$

$$\frac{d[\mathrm{Abp}2\cdot \mathrm{Ag}]}{dt}=k_{\mathrm{on},2}[\mathrm{Abp}][\mathrm{Abp}\cdot \mathrm{Ag}]-k_{\mathrm{off},2}[\mathrm{Abp}2\cdot \mathrm{Ag}]$$

$$\frac{d[\mathrm{Abp}]}{dt}=-k_{\mathrm{on},1}[\mathrm{Abp}][\mathrm{Ag}]+k_{\mathrm{off},1}[\mathrm{Abp}\cdot \mathrm{Ag}]-k_{\mathrm{on},2}[\mathrm{Abp}][\mathrm{Abp}\cdot \mathrm{Ag}]+\;k_{\mathrm{off},2}[\mathrm{Abp}2\cdot \mathrm{Ag}]$$

$$\frac{d[\mathrm{Ag}]}{dt}=-k_{\mathrm{on},1}[\mathrm{Abp}][\mathrm{Ag}]+k_{\mathrm{off},1}[\mathrm{Abp}\cdot \mathrm{Ag}]-k_{\mathrm{on},3}[\mathrm{Abp}\cdot \mathrm{Ag}][\mathrm{Ag}].$$

We simulated the time-dependent dynamics of relative concentration for each component in immunoagglutination. The concentrations of Abp·Ag and Abp2·Ag were sampled at a specific time point and normalized by initial Abp concentration, yielding normalized binding levels. Various rate constants (*k*) combinations were tested with parameter restrictions, as shown in Table S2. The Python code is shown in Code S2.

To further confirm the immunoagglutination binding model, we measured the particle sizes and zeta potentials of the mixtures used for interfacial tension and viscosity measurements using the Zetasizer Nano ZS90 (Malvern Instruments Ltd., Malvern, UK).

### Statistical analysis

Microsoft Excel (Microsoft, Redmond, WA, USA) was used to determine the significance values between the control and the sample with varying eNAMPT concentrations in diluted whole blood, plasma, and clinical plasma samples. A two-sample *t*-test was conducted, using unequal variances between the control and each sample concentration and a *P*-value of less than 0.05 as being significant.

## Results and discussion

### Horizontal flow modeling

**Fig. 2.**
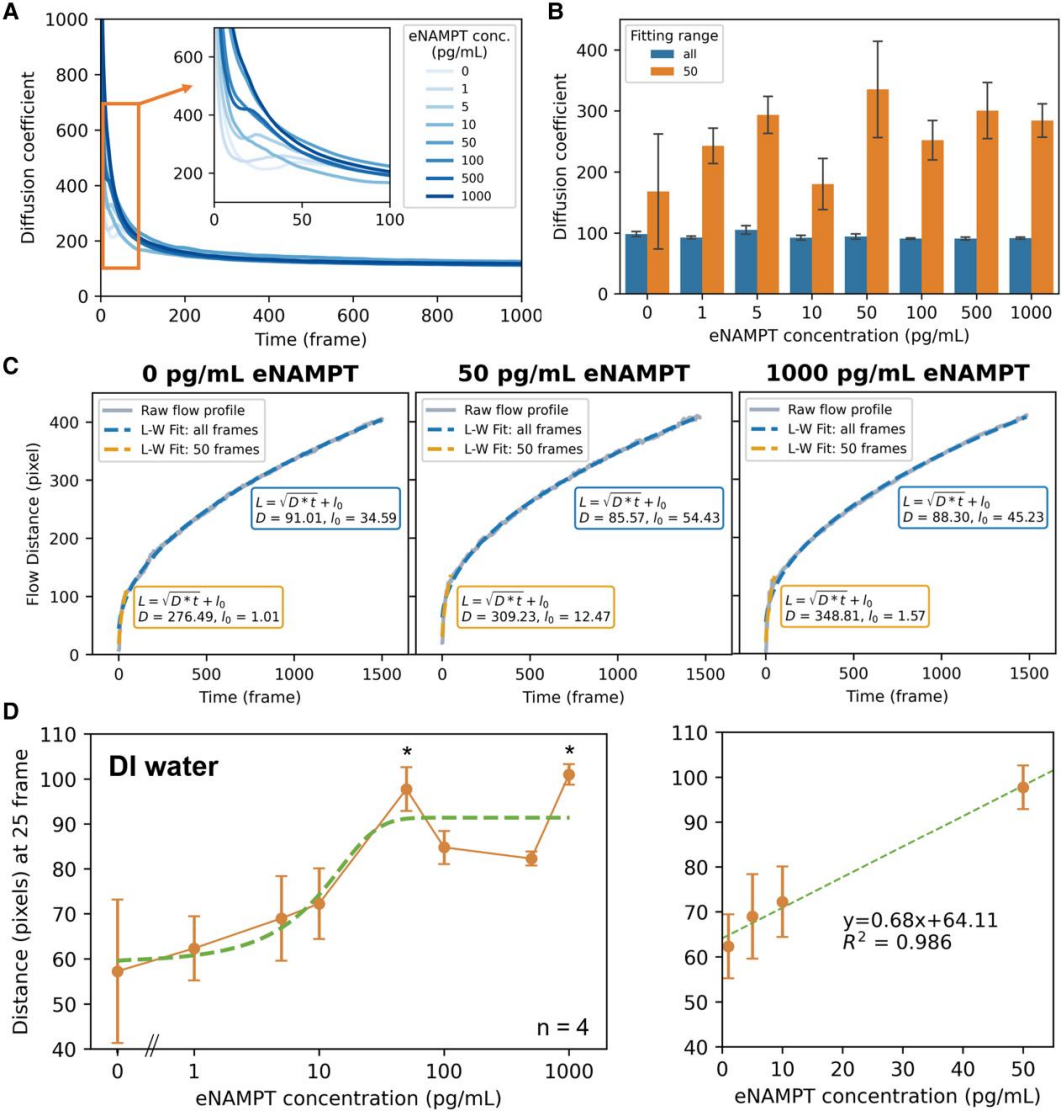
Horizontal flow modeling. A) The diffusion coefficient *D* from the L-W equation was computed at every time frame from the liquid movement *L* and time *t* plots (flow profiles) collected from the paper microfluidic chips. Moving averages were applied over 10 time frames. The main plot shows up to 1,000 frames (=33 s). The inset is a zoomed-in graph showing 0 to 100 frames (3.3 s). eNAMPT was dissolved in DI water, and the paper chips were preloaded with antibody-conjugated particles. B) Examples of raw flow profiles are shown with the L-W fitting with all (0–1,000) frames and 0–50 frames. C) Diffusion coefficient *D* was calculated from the L-W fittings with all frames and the first 50 frames. D) Flow distances at 25 frames (1 s) were used to represent the initial flow rate, plotted against the eNAMPT concentration. Averages from four experiments for each eNAMPT concentration (dissolved in DI water). A zoomed-in graph is shown on the right to indicate its linear range.

The flow rate-based assay with immunoagglutination was first investigated using a series of eNAMPT solutions in DI water. One microliter of anti-eNAMPT ALT-100 (mAb) conjugated particle suspension was preloaded to the inlet of each channel and followed by loading various eNAMPT solutions from 0 to 1,000 pg/mL in DI water. It is known that the capillary flow through a porous medium like cellulose paper should follow the L-W law mentioned above, where the liquid movement increases with the square root of time ($L=\sqrt{Dt}$). However, the liquid parameters, interfacial tension *γ*_LG_, contact angle *θ*, and dynamic viscosity *μ*, may change during the flow, resulting from the particles’ immunoagglutination and their retention in the paper pores. As such, the diffusion coefficient *D* may not be constant, leading to the deviation from the square root fitting. The flow dynamics were first analyzed by evaluating *L*^2^/*t* = *D*, calculated at every time point and utilizing moving averages. As shown in Fig. 2A, diffusion coefficients *D* were substantially high at the initial stage of the flow, gradually decreased as the flow proceeded further, and remained relatively constant after 100 frames (3.3 s). While no significant difference could be observed among eNAMPT concentrations after 100 frames, the diffusion coefficients at the initial stage before 50 frames (1.7 s) were substantially high and different among eNAMPT concentrations. We then made L-W fittings for the first 50 frames and all frames (0–1,500 frames) for each eNAMPT concentration, and the examples are shown in Fig. 2C. Higher diffusion coefficients *D* were observed at the first 50 frames than all frames (3.05, 3.61, and 3.95 times). The first 50 frames followed steep linear curves better, while the remainder followed the square root (L-W) fittings. Figure 2B shows the statistics of diffusion coefficient estimation for all eNAMPT concentrations. The flow's initial stage (first 50 frames) showed better discrimination among antigen concentrations than all-frame data. These results indicated that immunoagglutination could not affect the later flow stage; in other words, the level of immunoagglutination, i.e. the target eNAMPT concentration, was mainly determined at the initial flow stage. Therefore, we measured the flow distance *L* at 25 frames, the halfway point of the initial steep increase in the flow distance (= initial steep decrease in diffusion coefficient) for best discrimination among antigen concentrations, as shown in Fig. 2D. This flow distance at a particular time point (*L* at 25 frames) increased as eNAMPT concentrations rose, leveled off at a plateau, and decreased at very high concentrations, like a typical immunoassay. The linear range was 1–50 pg/mL. Suppose the sample is diluted at a factor of 100. In that case, the linear range of the undiluted sample should be 0.1–5 ng/mL, which is suitable for quantifying eNAMPT from healthy (1–2 ng/mL) individuals. The dilution factor of 1,000 corresponds to the undiluted linear range of 1–50 ng/mL, suitable for unhealthy levels (10–20 ng/mL) (4). Contrary to the previous work (17) and other paper-based immunoassays, the particles were not captured, and competing targets were neither preloaded nor immobilized. As a result, we should distinguish a subtle difference at the initial flow stage, as shown in the assay results.

Particle retention within the paper pores could be one of the underlying reasons for optimal discriminations during the initial flow. Antibody-conjugated particles would flow out from the inlet with sample loading; however, agglutinated particles with high eNAMPT levels would be left behind at some point, resulting from their increased size and complex morphology (Fig. 1A). The particle retention also caused the decrease of the diffusion coefficient in the flow propagation, as shown in the mathematical modeling. We acquired fluorescence microscopic images of aggregated particles on cellulose paper to validate the aggregated particles’ retention during the flow (Fig. S1). A single particle's diameter is about 500 nm, which is hard to image with smartphone-based fluorescence microscopy; the bright spots primarily represent the immunoagglutinated particles larger than 1 μm. The number of particle aggregations is small with 0 pg/mL eNAMPT (a small number can be considered nonspecific aggregation; Fig. S1A). We found many particle immunoagglutinations with 50 pg/mL eNAMPT, as shown in Fig. S1B. We also acquired images from various points in the channel (Fig. S1C), and the normalized numbers of aggregated particles were plotted against the distance from the inlet (Fig. S1D). Many aggregated particles with 20 and 1,000 pg/mL eNAMPT concentrations were found along the channel, while those with 0 pg/mL were minimal (Fig. S1D). Nonaggregated particles should saturate the liquid–gas interface (= wetting front), substantially lowering the interfacial tension *γ*_LG_ and subsequently the diffusion coefficient *D*. With the immunoagglutinated particles being trapped in the nitrocellulose paper and left behind, especially during the initial flow, *γ_LG_* and *D* should be substantially higher. Thus, with high eNAMPT concentration in the sample, immunoaggltuinated particles’ retention caused the increases in diffusion coefficient and the flow rate, confirming the hypothesis described above and the principal scheme illustrated in Fig. 1A.

### Parameter evaluations

**Fig. 3.**
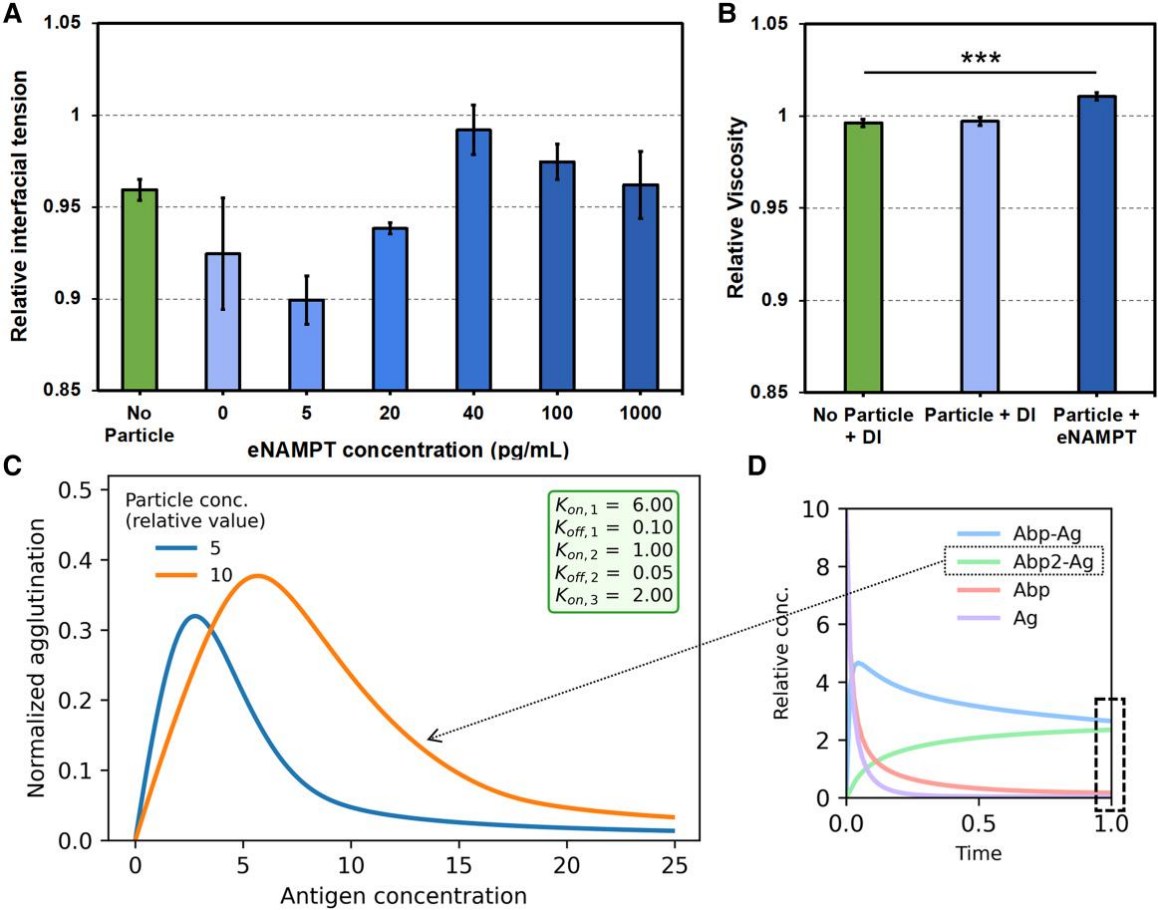
Parameter evaluations and immunoagglutination modeling. A) Interfacial tensions were measured by pendant drop analysis for the mixtures of varying eNAMPT concentrations + antibody-conjugated particles (*n* = 9). The ratio of the eNAMPT solution to the particle suspension and the particle concentration were identical to those of the main experiments. All interfacial tensions were normalized to DI water. “No particle” samples include neither particles nor eNAMPT and contain only buffers (hence slightly lower than the DI water). B) Viscosities were measured by an Ostwald viscometer (*n* = 4), normalized to DI water. The eNAMPT + particle mixtures used in the interfacial tension measurements were diluted by a factor of 50 to increase the volume needed for the Ostwald viscometer. C) The immunoagglutination level was simulated with varying the antigen (eNAMPT) concentrations and the particle concentration. The inset is the time course of the binding reaction between antibody-conjugated particle (Abp) and antigen (Ag). Abp–Ag indicates one particle bound with one antigen. Abp2–Ag indicates two particles bound with one antigen, which was used to simulate the immunoagglutination level. Rate constants were *k*_on,1_ = 6, *k*_off,1_ = 0.1, *k*_on,2_ = 1, *k*_off,2_ = 0.05, and *k*_on,3_ = 2. Other rate constant combinations are shown in Table S2, and their results are shown in Figs. S2 and S3. The binding was leveled off at *t* = 1, which was used to create the main binding plot.

The L-W equation indicates that the diffusion coefficient is a function of interfacial tension and viscosity, since $L=\sqrt{Dt}=\sqrt{\frac{R\gamma_{\mathrm{LG}}\cos\;\theta }{2\mu }t}$. The pore size *R* and contact angle *θ* were considered relatively constant based on the previously published findings (22). Therefore, interfacial tension *γ*_LG_ and viscosity *μ* were measured using a pendant drop method and a viscometer, using the antibody-conjugated particles and a series of eNAMPT solutions. The results are shown in Fig. 3A and B. The interfacial tensions at low eNAMPT concentrations (0–5 pg/mL) were lower than those with no particles, indicating that the particles (relatively hydrophobic and large sized) contributed to lowering the interfacial tension. With increasing eNAMPT concentrations, a notable trend emerged: the interfacial tension initially increased, reaching its maximum value at 40 pg/mL of eNAMPT before stabilizing at a similar level with higher eNAMPT concentrations. This observed trend closely corresponds with the assay results depicted in Fig. 2D, suggesting interfacial tension's predominant role in influencing capillary flow dynamics. Viscosities of the same solutions were also measured using an Ostwald viscometer, and no noticeable differences could be found (Fig. 3B). Therefore, interfacial tension is a predominant factor in influencing capillary flow rates.

### Immunoagglutination modeling

We also simulated the particle immunoagglutination level with varying particle concentrations. A series of differential equations were used, which were described in the immunoagglutination modeling section of materials and methods. While various combinations of rate constants *k* were tested, as summarized in Table S2, it resulted in similar trends, only being shifted in the x-axis (antigen concentration) (Figs. S2 and S3). Therefore, representative results are shown in Fig. 3C. These immunoagglutination levels (Abp2·Ag) were plotted against time (Fig. 3C on the right) or antigen (eNAMPT) concentration (Fig. 3C on the left). More time-dependent binding dynamics are shown in Fig. S2. All Abp2·Ag concentrations were normalized by initial Abp concentration, yielding normalized agglutination. Therefore, these simulations only show the trends, not the absolute numbers with a unit. From the time-dependent dynamics, *t* = 1 was chosen, where Abp·Ag (binding of one Abp and one Ag) and Abp2·Ag (binding of two Abp and one Ag) reached plateaus (Fig. 3C on the right and Fig. S2). At this time, the binding levels were simulated with varying particle (Abp) concentrations. Figure 3C shows these binding events, which initially increased with increasing antigen concentration, saturated at a plateau, and later decreased in the antigen-excess zone. It also shows a “shift” in the curve by changing the particle concentration. Such binding trends were further confirmed by measuring the particle size and zeta potential of antibody-particles + eNAMPT solution mixtures using the Zetasizer (Fig. S4). As expected, the particle size increased with increasing eNAMPT concentrations, and the zeta potential decreased. (Since the volume of antibody-conjugated particles was too small to be measured on the Zetasizer, we diluted the mixtures substantially. Therefore, the changes in particle size and zeta potential were not as significant as expected; these results should be used only to indicate the overall trends.)

In summary, higher eNAMPT concentrations induced more particle immunoagglutination, increasing the interfacial tension and contributing to a higher diffusion coefficient and quicker flow. Nitrocellulose fibers impeded the immunoagglutinated particles due to their increased size and higher chance of deposition. Such particle retention made particles with antigens lag away from the wetting front (liquid–gas interface). With fewer particles in the wetting front, the interfacial tension is close to the maximum, and the capillary flow rate becomes even higher.

### Assays with 10 and 1% whole human blood samples

**Fig. 4.**
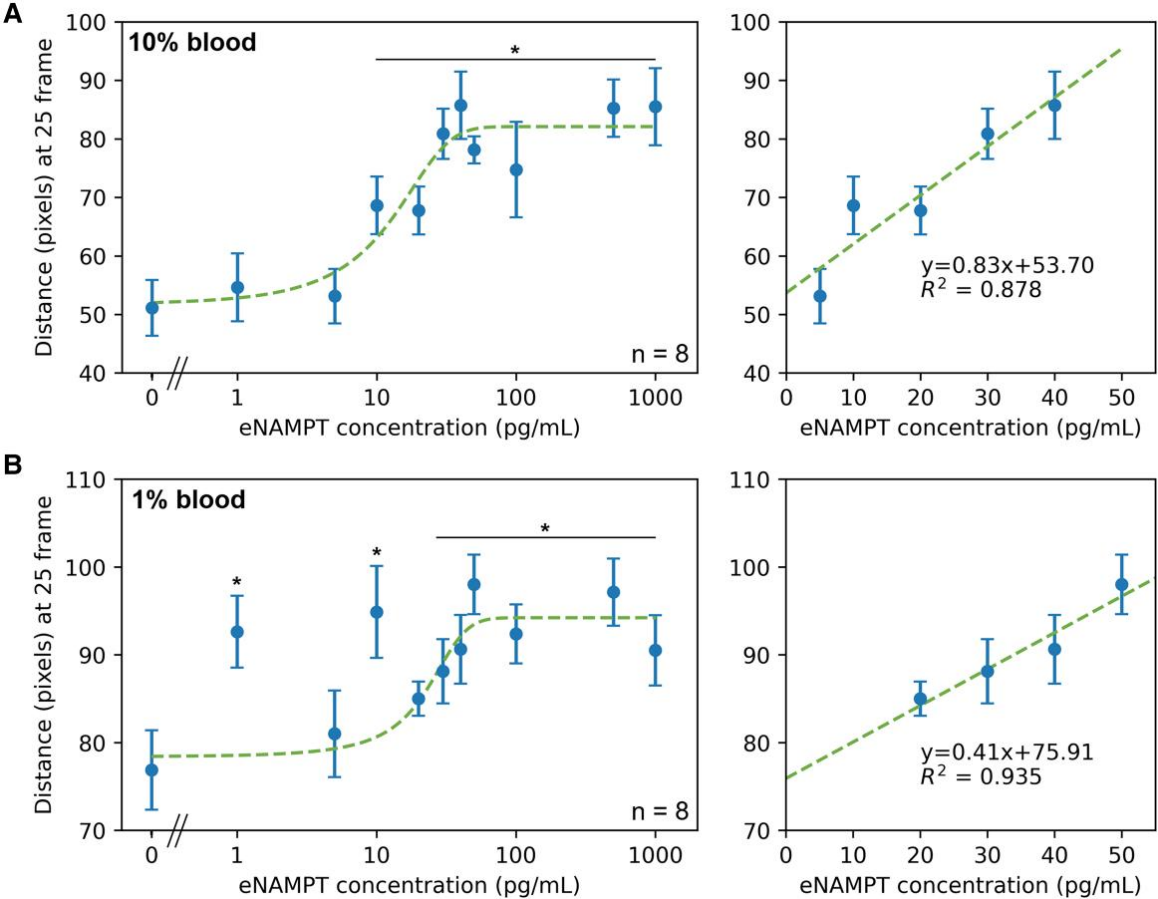
Assays with 10 and 1% whole human blood samples. A) Flow distances of 10% human blood samples were measured at the optimized time of 25 frames (left), and a zoomed-in graph showing the linear range (right). B) Flow distances of 1% human blood samples were measured at the optimized time of 25 frames (left), and a zoomed-in graph showing the linear range (right). All eNAMPT concentrations represent those in diluted samples. Averages and standard errors from eight experiments (two new paper chips and four channels per chip). * indicates significant differences from the negative control (no eNAMPT spiked to diluted blood) with *P* < 0.05.

The immunoagglutination flow assay was validated with eNAMPT spiked into 10 and 1% diluted human blood samples. eNAMPT (antigen) concentrations ranging from 0 to 1,000 pg/mL in 10% blood are equivalent to 0–10 ng/mL in whole blood, and those in 1% blood to 0–100 ng/mL in whole blood. The flow distances at the specific time point (25 frames as optimized in DI water results) were plotted against the eNAMPT concentrations in diluted whole blood (Fig. 4). (Figure S5 shows the individual flow distance profiles with 25 frames highlighted, indicating the optimized time from the DI water assays; they also made the satisfactory separation among different concentrations in blood assays.) Figure 4A shows assay results with 10% blood. The lowest concentration significantly different from the negative control (0 pg/mL eNAMPT spiked to 10% blood) was 20 pg/mL, the limit of detection (LOD) for this assay. This LOD corresponds to 0.2 ng/mL in whole human blood. The linear range for this assay is 5–40 pg/mL with an *R*^2^ of 0.878, as seen in Fig. 4A. Figure 4B shows the results with 1% blood samples. The LOD for this assay was 1 pg/mL, corresponding to 0.1 ng/mL in whole human blood. The linear range for this assay is 20–50 pg/mL with an *R*^2^ of 0.935, as seen in Fig. 4B. While minor differences between 10 and 1% whole blood assays could be observed, e.g. slightly lower LOD with 1% and marginally broader linear range with 10%, there is no fundamental difference between them. In addition, a data fluctuation was observed in lower eNAMPT concentrations (<10 pg/mL) with 1% whole blood assays (Fig. 4B). Therefore, 10% whole blood assay was deemed optimal and appropriate. It should also be noted that 100% whole blood could not be assayed, as it could not flow through the paper channels. For both dilutions, the flow distances slightly decreased at higher eNAMPT concentrations, indicating too many antigens (eNAMPT) for the available antibodies (anti-eNAMPT) in each paper chip channel, compromising antigen–antibody binding. This decrease at high concentrations follows the immunoagglutination model quite well, as shown in Fig. 3C.

A small amount of eNAMPT should still exist in the whole blood of healthy subjects. Up to 1–2 ng/mL of eNAMPT can be found in the undiluted plasma of healthy subjects (4). Converting these values to whole blood-equivalent concentrations and applying 10 and 1% dilutions would decrease these concentrations to 5–10 pg/mL, undetectable with typical ELISA. Nonetheless, actual eNAMPT concentrations would be higher than those shown in the x-axes of Fig. 4. These assays should be interpreted as detecting the “spiked” eNAMPT on top of the existing eNAMPT (e.g. control).

### Assays with 10 and 1% human plasma samples

**Fig. 5.**
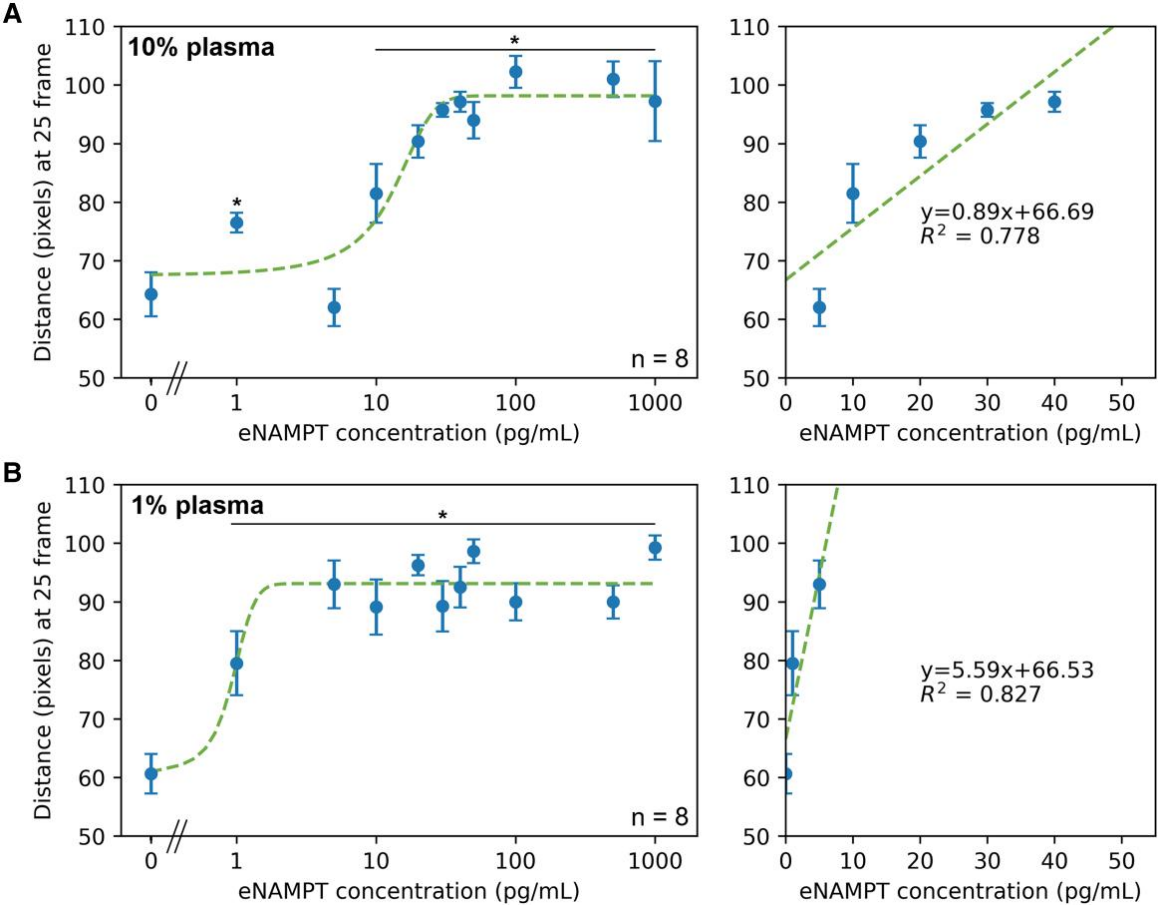
Assays with 10 and 1% human plasma samples. A) Flow distances of 10% human plasma samples were measured at the optimized time of 25 frames (left), and a zoomed-in graph showing the linear range (right). B) Flow distances of 1% human plasma samples were measured at the optimized time of 25 frames (left), and a zoomed-in graph showing the linear range (right). All eNAMPT concentrations represent those in the diluted samples. Averages and standard errors from eight experiments (two new paper chips and four channels per chip). * indicates significant differences from the negative control (no eNAMPT spiked to diluted blood) with *P* < 0.05.

An identical set of experiments was performed, while eNAMPT was spiked into 10 and 1% diluted human plasma samples. These 10 and 1% human plasma samples offer a better comparison to the clinical sample assays discussed in the following section. The same optimized time point, 25 frames from the DI water assays, was utilized to extract the flow distances (Fig. 5). The LOD for this assay was 10 pg/mL, corresponding to 0.1 ng/mL in whole human plasma. The linear range of this assay was from 5–40 pg/mL, with an *R*^2^ value of 0.778 (Fig. 5A). Experiments were repeated with 1% plasma samples (Fig. 5B). The LOD was 1 pg/mL, corresponding to 0.1 ng/mL in whole human plasma, i.e. identical to the 10% assay result. The linear range of this assay was 0–10 pg/mL (narrower than 10% results), with an *R*^2^ value of 0.827 (Fig. 5B). The LODs of plasma assays are comparable to or slightly lower than those of whole blood assays. Between 10 and 1%, the 10% plasma assays offered a broader linear range than the 1% plasma assays, while the whole plasma equivalent LODs were identical. Therefore, 10% plasma samples would present a better capability of distinguishing between the different eNAMPT concentrations spiked into the samples. It should also be noted that these assays detect the “spiked” eNAMPT on top of existing eNAMPT, as the pre-existing eNAMPT concentrations could not be quantified with typical ELISA.

### Assays with clinical samples

Finally, assays were conducted with clinical plasma samples from fifteen subjects. Of the fifteen clinical samples, their eNAMPT concentrations were distributed between 1 and 200 ng/mL, as determined by ELISA (26). The eNAMPT concentrations in healthy human subjects are up to 1–2 ng/mL (4) and are up to ∼200 ng/mL in unhealthy subjects, requiring the detection range of 2–200 ng/mL detection range. With 10% or 1% dilution, the detection range would be shifted to 0.2–20 ng/mL or 20–2000 pg/mL in diluted samples, which exceeded the 5–40 pg/mL range discovered in Fig. 5. This inference was further proved by experimental results, as shown in Fig. S6. Clinical samples all gave high responses versus negative control with no eNAMPT present; however, they could not be differentiated since they all exceeded the detection range. High eNAMPT concentrations induce high levels of particle immunoagglutination utilizing most free particles, resulting in nearly maximum responses observed with all clinical samples.

**Fig. 6.**
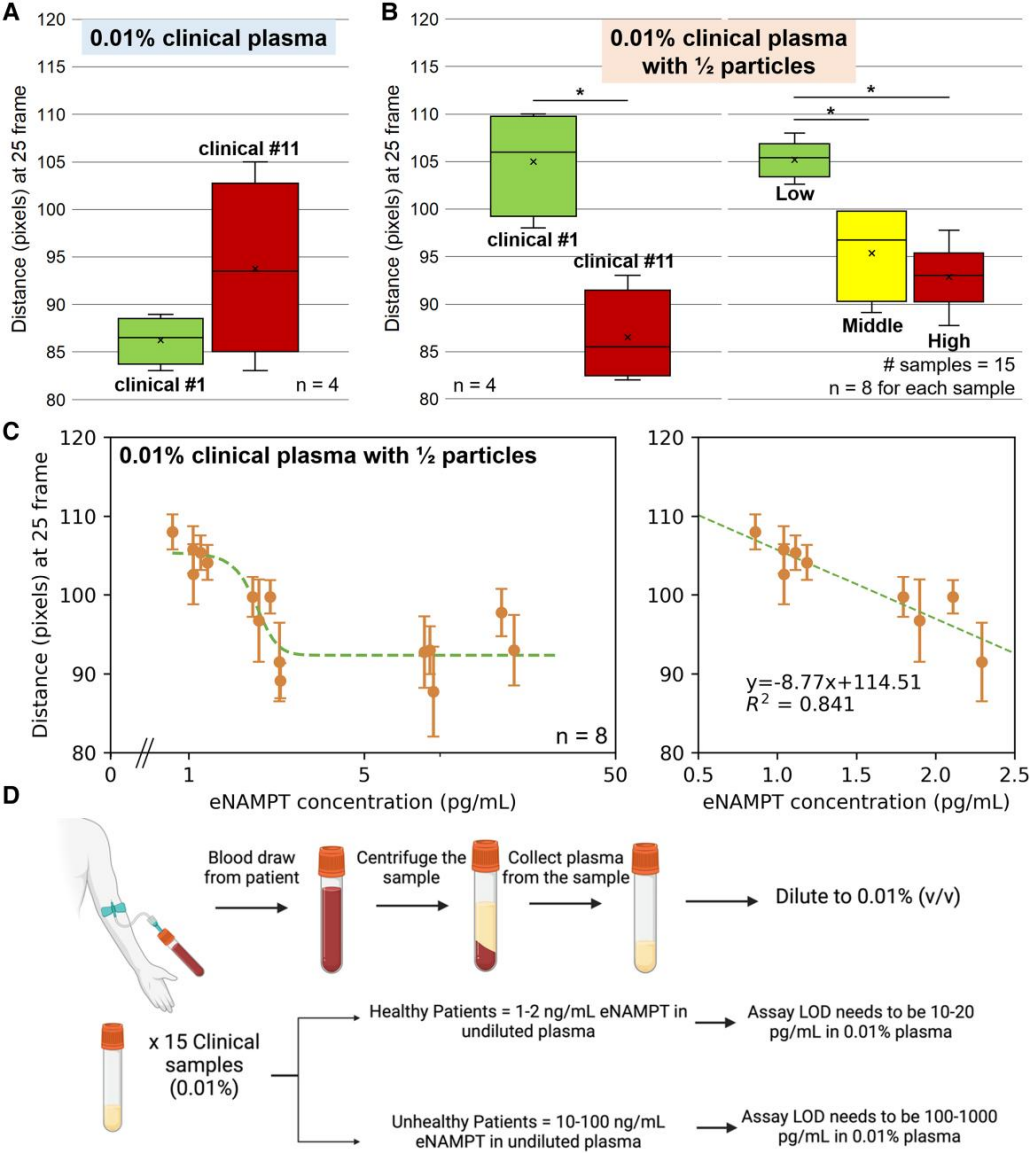
Validating the assay with 0.01% dilutions of clinical plasma samples. A, B) Box-and-whisker plots of the flow distances at the optimized time of 25 frames, using 0.01% clinical plasma samples. A) Comparison of clinical samples #1 (8.6 ng/mL) and #11 (86.8 ng/mL). Both samples were assayed four times (one paper chip per sample, each with four channels). B) Assay results using half antibody-conjugated particles. Left is the comparison of clinical samples #1 and #11 (each assayed four times); right is the comparison across all 15 clinical samples. * indicates significant differences among the groups (*P* < 0.05). Fifteen clinical samples were classified into three groups: low = 5 samples of 8.6–11.9 ng/mL, middle = 5 samples of 18.0–23.1 ng/mL, and high = 5 samples of 86.8–197 ng/mL. Each sample was assayed eight times (two paper chips per sample, each with four channels). Total number of assays = 120. C) The flow distances of all 15 clinical plasma samples are plotted against the ELISA-determined eNAMPT concentrations. Average and standard errors from eight different experiments (two paper chips per sample, each with four channels). D) Assay procedure for clinical samples. A blood sample is drawn from a patient, spun down to retrieve the plasma sample, and diluted to 0.01% (v/v). Necessary LODs are shown for diluted plasma to distinguish healthy and unhealthy patients.

Therefore, clinical samples were finally assayed with 0.01% dilution to shift the required detection range to 0.2–20 pg/mL, which resided in the same magnitude of LOD and linear range. Also, the antibody-conjugated particles were reduced to half. With these adjusted parameters, a better distinction was achieved between samples #1 and #11 (Fig. 6B on the left) compared to the results using the original particle concentration (Fig. 6A). The eNAMPT concentrations of samples #1 and #11 in undiluted plasma were 8.6 and 86.8 ng/mL, respectively, representing low and high concentrations. The assay results across all 15 clinical samples are shown in Fig. 6B on the right. Statistical distinctions could be made between low (8.6–11.9 ng/mL) and middle (18.0–23.1 ng/mL) concentrations (*P* < 0.05) and between low and high (86.8–197 ng/mL) concentrations (*P* < 0.05).

Interestingly, the distances were higher with the lower concentrations, i.e. a flipped trend. The lower number of antibody-conjugated particles could induce sufficient particle immunoagglutination with lower eNAMPT concentrations, leading to minimal particles at the wetting front and, subsequently, higher flow distances. At higher eNAMPT concentrations, antibody–antigen binding was no longer preferred as the antigen concentrations were too high for one-half antibody concentration. We have already observed decreases in higher eNAMPT concentrations after the peak points in Figs. 4 and 5. Such a flipped trend was also supported by the immunoagglutination model (Fig. 3C). With a lower concentration of antibody-conjugated particles (blue line with Abp = 5), the curve shifted to the left, compared to a higher particle concentration (orange line with Abp = 10). This shift gave a cross-section of two curves, where the assay with more particles showed an upward trend while the assay with fewer particles showed a downward trend.

The flow distances with 0.01% clinical plasma and one-half particle concentration were plotted against the eNAMPT concentrations (quantified with ELISA), as shown in Fig. 6C. A good linearly decreasing trend could be observed from 0.86–2.3 pg/mL (equivalent to 8.6–23 ng/mL in undiluted plasma). The developed assay can identify whether a sample falls into the low range, i.e. close to a healthy status, or into the middle-high range, requiring antibody administration to treat ARDS.

### Long-term stability of wax-printed chips

If wax printer paper chips were left in an open box at room temperature for a long time, the wax patterns would deteriorate, resulting in sharp spike patterns at the wetting front (instead of parabolic shape) or even penetrating the channel boundaries. In such a case, the resulting flow profiles would not follow the square root of the time trend, i.e. the L-W law. When both unprinted papers and wax-printed chips were stored in a sealed plastic container or bag at 4°C refrigerator, we did not observe such issues for at least 4 months, during which time we collected all the data shown in this manuscript.

## Conclusion

This work demonstrates the ability to detect various concentrations of eNAMPT present in human blood and plasma samples, including clinical plasma samples. It classifies the eNAMPT concentrations into low (close to normal), middle, and high. Overall, this work aims to demonstrate quick, cost-effective, and easy-to-use detection of eNAMPT from human blood and plasma samples. Noncompetitive particle immunoagglutination was conducted on paper microchannels, and their extents were quantified with the capillary flow rates (as represented by the flow distances) on paper chips. While particles were not “captured” on a paper substrate as they did in competitive immunoassays, we could still identify a subtle difference at the liquid–gas interface, i.e. wetting front, resulting from particle immunoagglutination. Flow rate (distance) was measured by a smartphone in a rapid and easy-to-use manner. Cloud-based Google Colab automatically analyzed the flow distance from each paper microchannel. The time points were optimized for different types and dilutions of samples using the horizontal flow model and the immunoagglutination binding model. The horizontal flow model justified the use of the first 25 frames (ca. 1 s) in maximally differentiating the eNAMPT concentrations. Interfacial tensions and dynamic viscosities were also measured for the assay mixtures to describe their contributions to the horizontal flow model. The LODs were 10–20 pg/mL in 10% whole blood and 10% plasma, equivalent to 0.1–0.2 ng/mL in undiluted blood and plasma. With 1% blood and plasma, the LOD was 1 pg/mL, equal to 0.1 ng/mL in undiluted blood and plasma. The linear range was 5–40 pg/mL with 10% blood and plasma. Clinical samples should be diluted to 0.01%, and the antibody-particle concentration should be reduced to half, resulting in a successful distinction between low and middle-to-high eNAMPT concentrations. The immunoagglutination binding model explained the need for such dilutions, the “shifts” in the assay curves, and the “flipped” trends in clinical plasma samples. As mentioned earlier, this POC test is beneficial to the potential success of implementing anti-eNAMPT as drug therapy in patients with ARDS by offering a quick and straightforward method to detect if eNAMPT levels are high enough to warrant the administration of the drug. In the future, this work should be automated further as a POC test that could be conducted in the patient's home quickly and straightforwardly. This work demonstrates the ability to detect eNAMPT concentration levels in clinical plasma samples using cheap materials and a simple assay model while still providing high analytical sensitivity (very low LOD).

## Supplementary Material

pgae173_Supplementary_Data

pgae173_Supplementary_Data

## Data Availability

Protocol for covalent coupling of antibodies to the submicron particles is available at protocols.io: https://doi.org/10.17504/protocols.io.bhsvj6e6. Protocol for wax printing the paper-based microfluidic chips is available at protocols.io: https://doi.org/10.17504/protocols.io.btqxnmxn. All data associated with this study are presented in the article and the supplementary materials.
