# Supplementary material for: Development of a cloud-based flow rate tool for eNAMPT biomarker detection
Source: PNAS Nexus. 2024 Apr 24;3(5):pgae173. doi: 10.1093/pnasnexus/pgae173 (PMC11071447; doi:10.1093/pnasnexus/pgae173)
Supplement: pgae173_Supplementary_Data [file pgae173_supplementary_data.pdf]

SUPPLEMENTARY MATERIALS

Development of a Cloud-Based Flow Rate Tool for eNAMPT Biomarker Detection

Bailey C. Buchanan, Yisha Tang, Hannah Lopez, Nancy G. Casanova, Joe G. N. Garcia, and Jeong-Yeol Yoon

TABLE OF CONTENTS

Supplementary Table S1. Characteristics of ARDS subjects (*n* = 15).

Supplementary Table S2. List of rate constant (*k*) combinations used in immunoagglutination modeling.

Supplementary Figure S1. Fluorescence microscopic images of the paper chips showing the extent of particle immunoagglutination and particle diffusivity.

Supplementary Figure S2. The time-dependent dynamics of relative concentration for each component in immunoagglutination.

Supplementary Figure S3. Immunoagglutination binding simulation results with varying rate constants *k*.

Supplementary Figure S4. Particle size and zeta potential of antibody-conjugated particles mixed with 0, 20, 1000 pg/mL eNAMPT solutions (*n* = 3 each).

Supplementary Figure S5. Representative raw flow distance profiles of the assays with 10% whole blood (A) and 10% plasma (B).

Supplementary Figure S6. Clinical assays with 10% and 1% dilutions.

Supplementary Code S1. Python code on Google Colab for flow rate profile analysis.

Supplementary Code S2. Python code for immunoagglutination modeling.

Supplementary Table S1. Characteristics of ARDS subjects (n=15).

Age (mean / SD)	53.4 / 18
Gender (male %)	53
Race (%)	
White	86.7
Black	20
Mortality (%)	60

Supplementary Table S2. List of rate constant (*k*) combinations used in immunoagglutination modeling. Total number of combinations = 45.

$k_{on,1}$	$k_{off,1}$	$k_{on,2}$	$k_{off,2}$	$k_{on,3}$	$k_{on,1}/k_{off,1}$	$k_{on,2}/k_{off,2}$
2	0.1	0.5	0.05	1	20	10
2	0.1	0.5	0.5	1	20	1
2	0.1	1	0.05	1	20	20
2	0.1	1	0.5	1	20	2
2	0.4	0.5	0.5	1	5	1
2	0.4	1	0.5	1	5	2
2	1	0.5	0.5	1	2	1
2	1	1	0.5	1	2	2
6	0.1	0.5	0.05	1	60	10
6	0.1	0.5	0.05	2	60	10
6	0.1	0.5	0.5	1	60	1
6	0.1	0.5	0.5	2	60	1
6	0.1	1	0.05	1	60	20
6	0.1	1	0.05	2	60	20
6	0.1	1	0.5	1	60	2
6	0.1	1	0.5	2	60	2
6	0.4	0.5	0.05	1	15	10
6	0.4	0.5	0.05	2	15	10
6	0.4	0.5	0.5	1	15	1
6	0.4	0.5	0.5	2	15	1
6	0.4	1	0.5	1	15	2
6	0.4	1	0.5	2	15	2
6	1	0.5	0.5	1	6	1
6	1	0.5	0.5	2	6	1
6	1	1	0.5	1	6	2
6	1	1	0.5	2	6	2
10	0.1	0.5	0.05	1	100	10
10	0.1	0.5	0.05	2	100	10
10	0.1	0.5	0.5	1	100	1

10	0.1	0.5	0.5	2	100	1
10	0.1	1	0.05	1	100	20
10	0.1	1	0.05	2	100	20
10	0.1	1	0.5	1	100	2
10	0.1	1	0.5	2	100	2
10	0.4	0.5	0.05	1	25	10
10	0.4	0.5	0.05	2	25	10
10	0.4	0.5	0.5	1	25	1
10	0.4	0.5	0.5	2	25	1
10	0.4	1	0.05	1	25	20
10	0.4	1	0.05	2	25	20
10	0.4	1	0.5	1	25	2
10	0.4	1	0.5	2	25	2
10	1	0.5	0.05	1	10	10
10	1	0.5	0.05	2	10	10
10	1	0.5	0.5	1	10	1
10	1	0.5	0.5	2	10	1

Note. The following restrictions were applied:

$k_{on,1}/k_{off,1} \geq k_{on,2}/k_{off,2}$ and $k_{on,1}/k_{off,1} \geq k_{on,3}$ – the equilibrium constant (= association over dissociation rates) of the primary binding event is greater than the secondary binding event.

$k_{on,1} \geq k_{on,2}$ and $k_{on,1} \geq k_{on,3}$ – the primary association rate constant is greater than the secondary association rate constants.

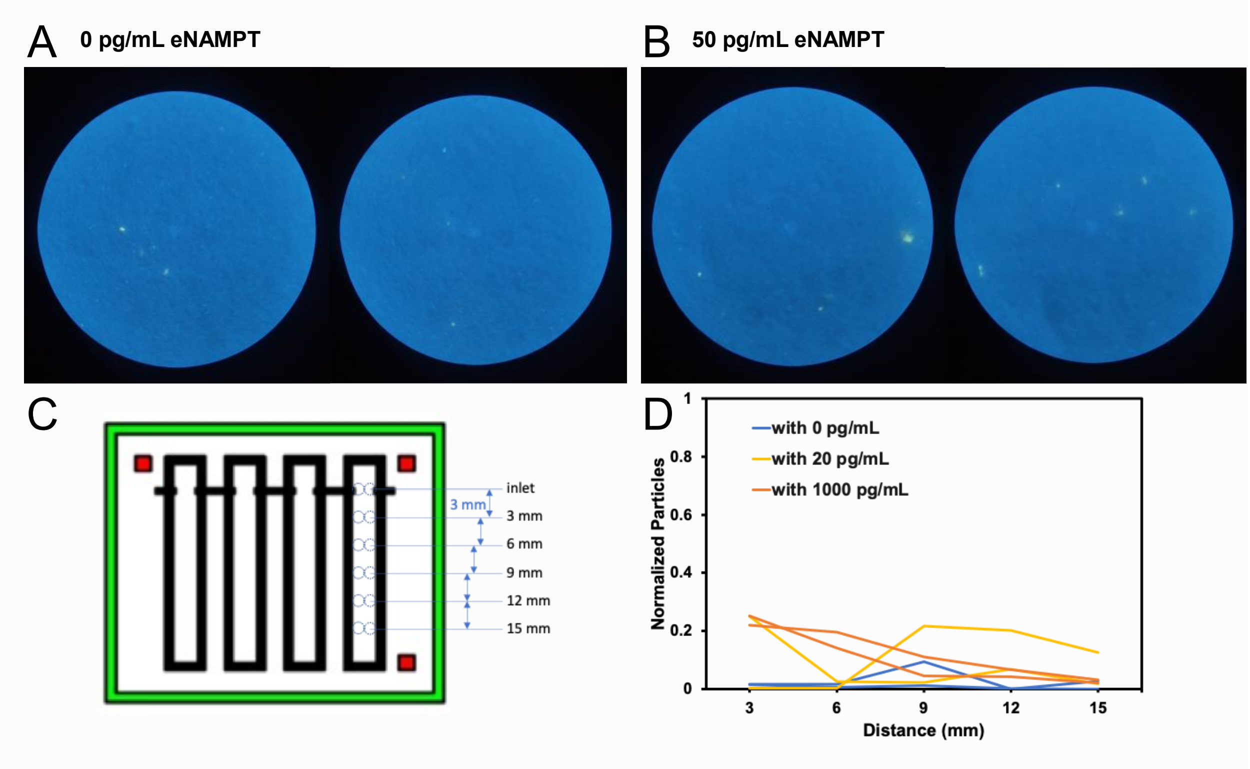

Supplementary Figure S1. Fluorescence microscopic images of the paper chips showing the extent of particle immunoagglutination and particle diffusivity. Both (A) and (B) are pre-loaded with 0.0318 µg/µL of anti-eNAMPT conjugated particles. (A) Images of particles after DI water sample with 0pg/mL concentration of eNAMPT added to the inlets. (B) Images of particles after DI water sample spiked with 50 pg/mL concentration of eNAMPT added to the inlets. (C) Dimensions of fluorescent imaging spots for (D). (D) Deposited particle amount along the microfluidic channel on paper chip (3 mm away from inlet to 15 mm away). Particle areas were analyzed by ImageJ and normalized by the total particle area found from all images of one entire channel.

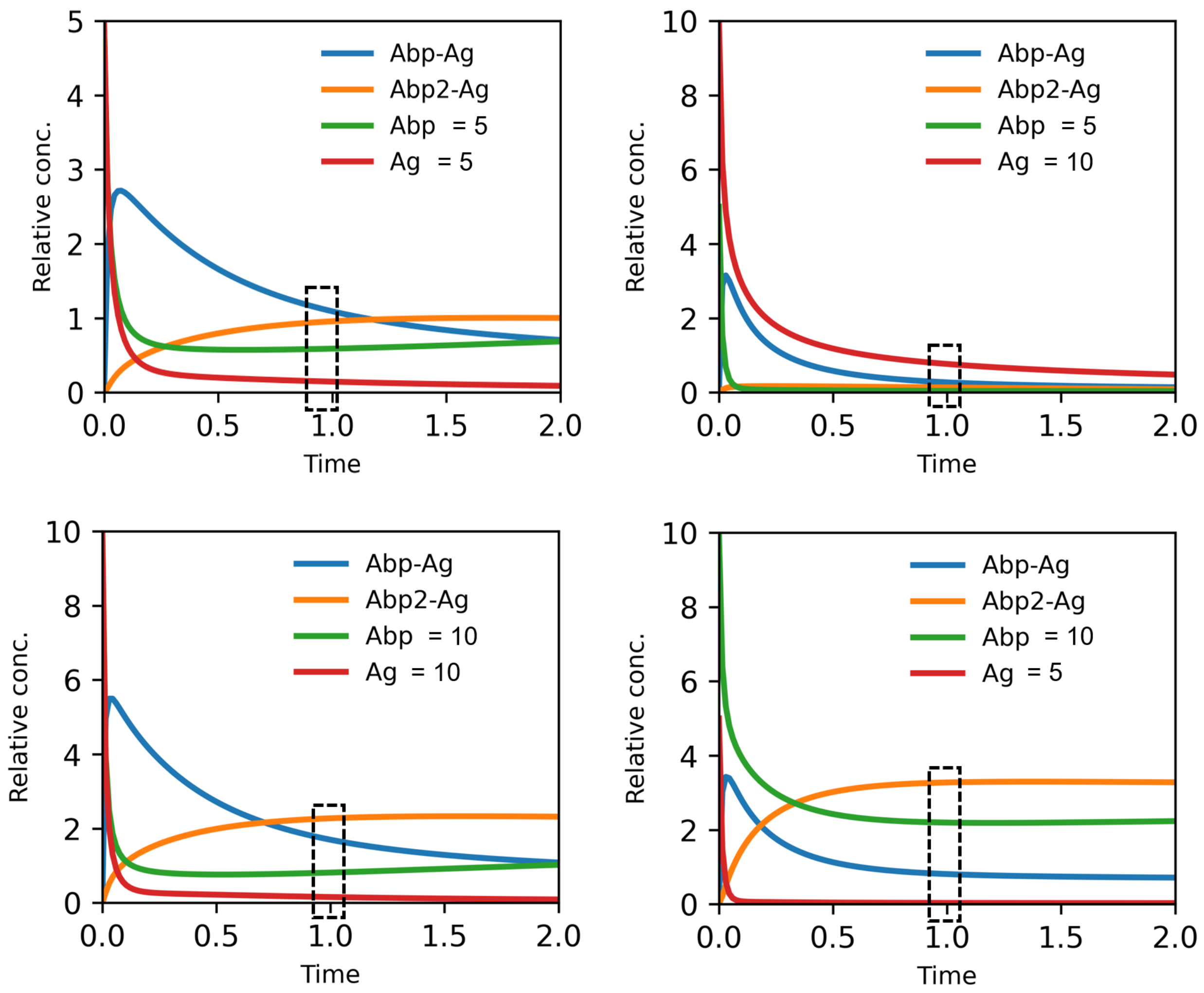

Supplementary Figure S2. The time-dependent dynamics of relative concentration for each component in immunoagglutination. Each figure represents the result with different initial amounts of antibody-conjugated particles and antigens introduced in the system. Rate constants were fixed at $k_{on,1}$ = 6, $k_{off,1}$ = 0.1, $k_{on,2}$ = 1, $k_{off,2}$ = 0.05, and $k_{on,3}$ = 2. t = 1 was used to generate Figure 3C and Supplementary Figure S3.

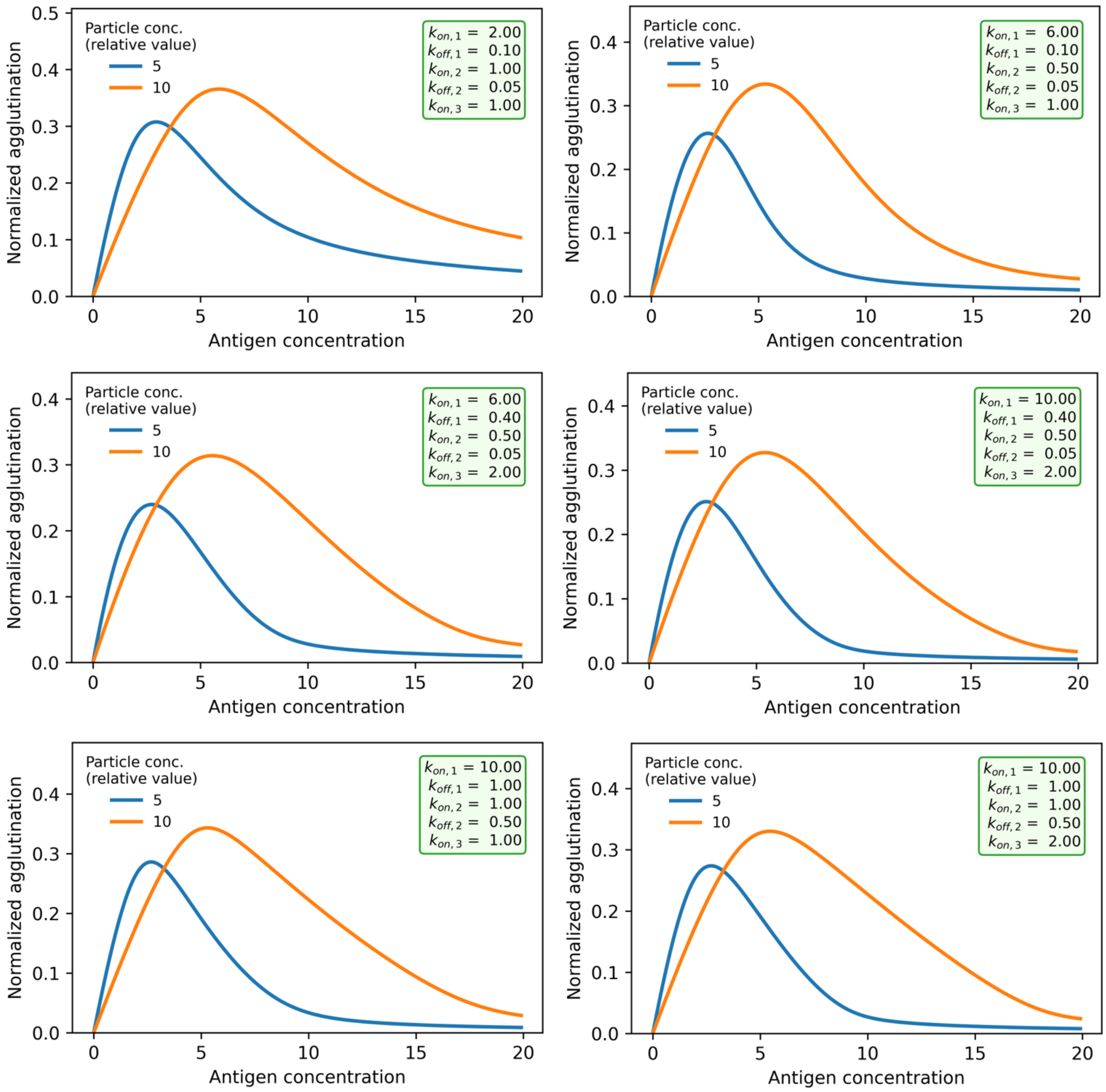

Supplementary Figure S3. Immunoagglutination binding simulation results with varying rate constants *k*. Each figure represents the result with different initial amounts of antibody-conjugated particles and antigens introduced in the system. Six combinations of rate constants are shown, all showing similar trends. There were 45 combinations, as shown in Supplementary Table S2.

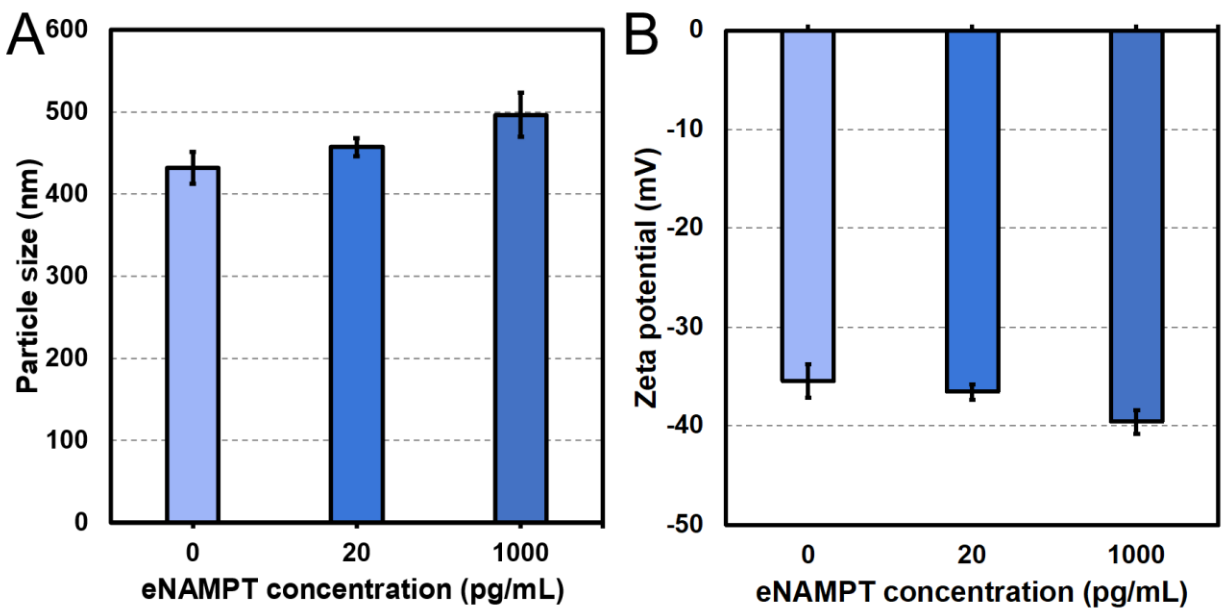

Supplementary Figure S4. Particle size and zeta potential of antibody-conjugated particles mixed with 0, 20, 1000 pg/mL eNAMPT solutions (n = 3 each). The mixtures were diluted by a factor of 4 to reach a suitable volume for measurement in the Zetasizer, indicating that the results could not directly infer the level of agglutination in the assays shown in the manuscript.

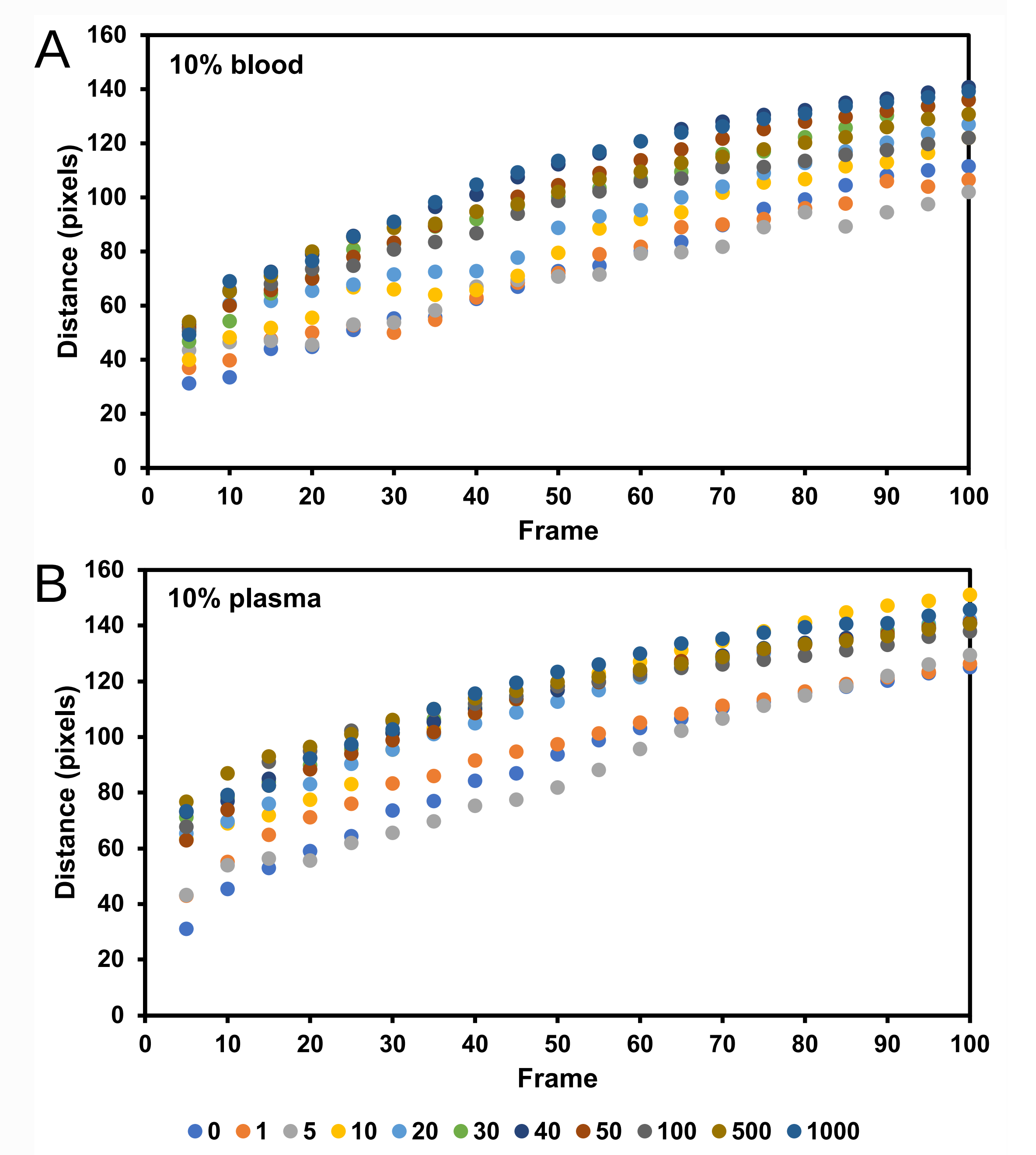

Supplementary Figure S5. Representative raw flow distance profiles of the assays with 10% whole blood (A) and 10% plasma (B). The bottom legend represents the eNAMPT concentration in pg/mL. Flow distances at 25 frames (optimized time point by DI water assay) are used.

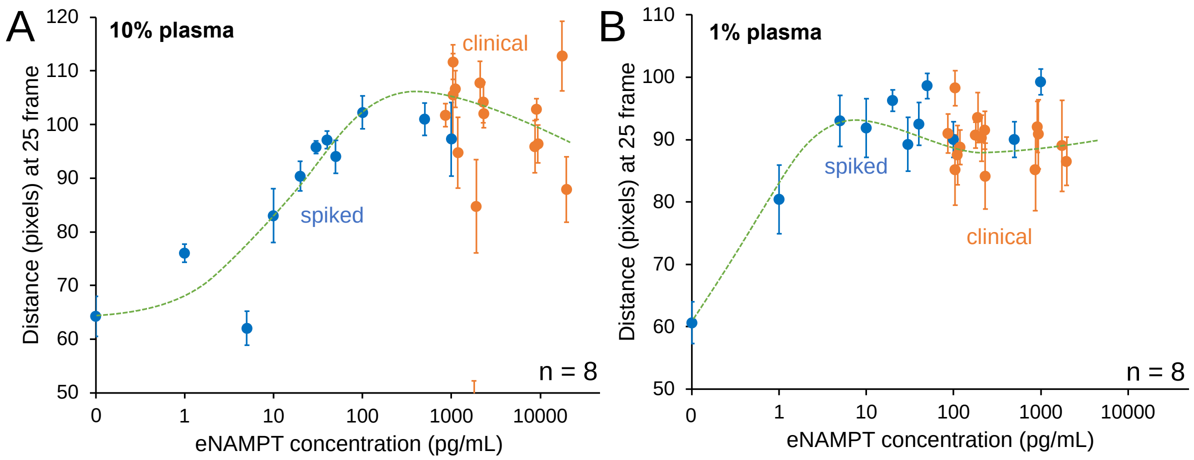

Supplementary Figure S6. Clinical assays with 10% and 1% dilutions. (A) Flow distances of 10% clinical (orange) and spiked (blue) plasma samples at the optimized time of 25 frames. (B) Flow distances of 1% clinical (orange) and spiked (blue) plasma samples at the optimized time of 25 frames. All eNAMPT concentrations represent those in the diluted samples. Averages and standard errors from 8 experiments using two new paper chips.

Supplementary Code S1. Python code on Google Colab for flow rate profile analysis.

```
# -*- coding: utf-8 -*-"""FlowCode-AlexLane-PFAS.ipynb

Automatically generated by Colaboratory.

Original file is located at
    https://colab.research.google.com/drive/16ytiELUX_ObbAp-
Op1ocIiDhwGpCqWfm

##**Flow Rate Profile Analysis**
Feb 2022 - Lane Breshears, modified from .py code Alex Day

Use with any microfluidic chip, place the black line over the channel to
analyze flow across the channel. Specify number of channels and channel
gaps.

## Setup

**Link CoLab code with your Google Drive**
"""

from google.colab import drive
drive.mount('/content/drive')

#
https://drive.google.com/drive/folders/1FWndAYiDDbhP_JWq7UcodWYYF2mCsjqb?u
sp=sharing

"""**Link CoLab code with Google Sheets**"""

from google.colab import auth
auth.authenticate_user()

import gspread
from google.auth import default
creds, _ = default()

gc = gspread.authorize(creds)

"""**Open the active folder with videos**"""

# Change the below to open up the folder that has the videos in it
!ls '/content/drive/MyDrive/PFOA_flowrate/0719eww'

"""**Import Libraries**"""

from tensorflow.python.util.tf_export import get_canonical_name_for_symbol
import numpy as np
import cv2
import os
import csv
import matplotlib.pyplot as plt
import sys
```

```
from google.colab.patches import cv2_imshow #to see images, can be deleted
later
import pandas as pd

import gspread_dataframe as gd
import gspread as gs

"""# Analyze videos"""

def createSpreadsheet(videoName):
      '''
      videoName = the name of the video being analyzed will be
      used to create the worksheet
      '''
      gc = gspread.authorize(creds)
      sh = gc.create(str(videoName))
      print(videoName)
      return sh

def defineLane(start, end):
      '''
      Auxilliary function used to correctly determine which pixels to look
at in the lane, regardless of whether the pixel
      numbers go from high to low or low to high
      '''
      points = []
      temp = end - start
      if temp < 0:
            for i in range(end, start):
                  points.append(i)
      else:
            for i in range(start, end):
                  points.append(i)
      return points

def flowAnalysis(videoName, location, start, end, channel, axes,
threshold=13):
      '''
      Given a video file, return a list of the flow profile of the moving
front. Each index will represent a frame of the
      original video file, and its value will represent how many pixels
the wetting front has flowed through

      Parameters:
      "videoName" = the file name of the video file
      "Location" = if the axis is horizontal, then location is the pixel
row containing the desired lane, while if the
                         axis vertical, then location is the pixel column
contained the desired lane
      "start" = the starting pixel in the row/column (dictated by
"Location" parameter) of the desired lane
      "end" = the ending pixel in the row/column (dictated by the
"Location" parameter) of the desired lane
```

```
	"axes" = determines whether the desired flow lane is horizontal or 
vertical in the video file (MUST BE EITHER "H" OR "V")
	"channel" = the channel number on the chip
	'''

	# NOTE: if you're not seeing any movement in your data, change this 
threshold value! It represents the brightness change
	# in a pixel within the flow lane that is considered to be high 
enough to conclude that the moving front has moved to
	# that location.
	threshold = threshold

	possibleAxes = ['H', 'V']
	if axes not in possibleAxes:
		print('Axes parameter not allowed, must be either H or V')
		sys.exit()
	laneLength = defineLane(start, end)

	flowProfile = []
	video = cv2.VideoCapture(videoName)
	print('Video name: {}'.format(videoName))
	print('Video length (frames): 
{}'.format(video.get(cv2.CAP_PROP_FRAME_COUNT)))
	success = True
	imageNumber = 1
	while success:
		# print('Image Number: {}'.format(imageNumber))
		success, image = video.read()
		if success:
			gray = cv2.cvtColor(image, cv2.COLOR_BGR2GRAY)
			if imageNumber == 1:
				initialLane = []
				for i in laneLength:
					triplet = []
					for j in range(-1, 2):
						if axes == 'H':
							triplet.append(gray[location + j, 
i])
						elif axes == 'V':
							triplet.append(gray[i, location + 
j])
					initialLane.append(np.average(triplet))

			currentLane = []
			differences = 0
			for i in laneLength:
				triplet = []
				for j in range(-1, 2):
					if axes == 'H':
							triplet.append(gray[location + j, 
i])
					elif axes == 'V':
						triplet.append(gray[i, location + j])
				currentLane.append(np.average(triplet))
```

```
                for i in range(0, len(laneLength)):
                    difference = initialLane[i] - currentLane[i]
                    if difference >= threshold:
                        differences += 1

                flowProfile.append(differences)
                imageNumber += 1



        print('Maximum flow (pixels): {}'.format(np.amax(flowProfile)))
        videoFPS = video.get(cv2.CAP_PROP_FPS)
        print('VIDEO FPS IS:')
        print(videoFPS)
        videoDuration = int(video.get(cv2.CAP_PROP_FRAME_COUNT)) / videoFPS
        print('Total Velocity: {:2f}'.format(float(np.amax(flowProfile) /
videoDuration)))
        print('------------------')

        plt.figure()
        plt.suptitle('Flow Profile (Pixels vs. Frame Number)')
        plt.plot(flowProfile)
        plt.show()
        return flowProfile

def exampleBounds(videoName, location, start, end, axes='H'):
        '''
        Given a video file, the function will save a grayscale image
depicting where the analysis algorithm would consider
        the lane to be (by using a solid black line over the first frame of
the video)

        Parameters:
        "videoName" = the file name of the video file
        "Location" = if the axis is horizontal, then location is the pixel
row containing the desired lane, while if the
                        axis vertical, then location is the pixel column
contained the desired lane
        "start" = the starting pixel in the row/column (dictated by
"Location" parameter) of the desired lane
        "end" = the ending pixel in the row/column (dictated by the
"Location" parameter) of the desired lane
        "axes" = determines whether the desired flow lane is horizontal or
vertical in the video file (MUST BE EITHER "H" OR "V")
        '''
        possibleAxes = ['H', 'V']
        if axes not in possibleAxes:
            print('Axes parameter not allowed, must be either H or V')
            sys.exit()
        laneLength = defineLane(start, end)
        video = cv2.VideoCapture(videoName)
        print(videoName)
        success = True
```

```
      success, image = video.read()
      gray = cv2.cvtColor(image, cv2.COLOR_BGR2GRAY)
      for i in laneLength:
            if axes == 'H':
                  gray[location, i] = 0
            elif axes == 'V':
                  gray[i, location] = 0
      cv2.imwrite('test bounds.png', gray)
      cv2_imshow(gray)


if __name__ == '__main__':

      #EDITABLE FOR DATA COLLECTION
      os.chdir(os.path.join(r'/content/drive/MyDrive/PFOA_flowrate/0719eww
'))  #C:\Users\owner\Downloads'))


      videoName = '20220719_102347.mp4'
      start = int(635) #100 vertical positioning)
      end = int(1105) #510 vertical positioning)
      channel_gap = int(138) #130 - cellulose jump from channel to chnl in
px distance
      location1 = int(806) #Row/channel initial position for horizontal
chip/Verticla chip would be column (moves horizontally)
      numberChannels = 4 #5 for cellulose - 4 for covid
      axes = 'H' #H - horizontal, V - vertical
      #Save the data to google sheets
      gc = gspread.authorize(creds)
      sh = gc.create(str(videoName))

      #END OF EDITABLE


      for i in list(range(0,numberChannels)):
            channel = i + 1
            if axes == 'H':
                  channel_location = location1 - channel_gap*(i) #assuming
1st channel is on bottom of chip
            elif axes == 'V':
                  channel_location = location1 + channel_gap*(i) #assuming
1st channel is on far left of chip
            else:
                  print('Check which channel you are starting on')

            print(channel_location)
            exampleBounds(videoName, channel_location, start, end, axes)
            flowProfile_list = flowAnalysis(videoName, channel_location,
start, end, i, axes, threshold=9)
            df_flowProfile = pd.DataFrame(flowProfile_list)

            ws = sh.add_worksheet(title=str(channel), rows="100",
cols="20")
            ws = gc.open(str(videoName)).worksheet(str(channel))
```

```
            gd.set_with_dataframe(ws, df_flowProfile)

"""Check your data"""

'''
#Click to go directly to the data collection spreadsheet of the video
analyzed - use latest version and make sure it has all the data
#Each sheet is a channel

Go to https://sheets.google.com to see your new spreadsheet
'''
```

Supplementary Code S2. Python code for immunoagglutination modeling. Also available at GitHub: https://github.com/yishat/ImmunoagglutinationModel


```
{ "cells": [
  {
   "cell_type": "markdown",
   "id": "0acf0a64-b62f-419b-a49b-e882c530ed94",
   "metadata": {
    "tags": []
   },
   "source": [
    "# **Setups**"
   ]
  },
  {
   "cell_type": "markdown",
   "id": "aa947360-2ad6-468c-ae84-9b43176d574c",
   "metadata": {
    "tags": []
   },
   "source": [
    "## Library"
   ]
  },
  {
   "cell_type": "code",
   "execution_count": null,
   "id": "e62ba3c7-6df7-4bbb-8c7f-ba6c4728d5fa",
   "metadata": {
    "tags": []
   },
   "outputs": [],
   "source": [
    "import numpy as np\n",
    "import pandas as pd\n",
    "import matplotlib.pyplot as plt\n",
    "from scipy.integrate import solve_ivp\n"
   ]
  },
  {
   "cell_type": "markdown",
   "id": "5d71b1b3-fa74-4129-9112-288a9c8da5e9",
   "metadata": {},
   "source": [
    "## Functions: agglutination model"
   ]
  },
  {
   "cell_type": "code",
   "execution_count": null,
   "id": "171115a4-2957-47aa-babe-713c32dad36f",
   "metadata": {
    "tags": []
```

```
   },
   "outputs": [],
   "source": [
    "# Differential equations describing the relationship of components in
the immunoagglutination system\n",
    "def Agglutination4_5(t, z, ko1, kd1, ko2, kd2, ko3):\n",
    "    # 4 components\n",
    "    # 5 k parameters\n",
    "    AbAg,Ab2Ag,Ab,Ag = z\n",
    "    return [+ko1*Ab*Ag-kd1*AbAg-ko2*Ab*AbAg+kd2*Ab2Ag-ko3*Ag*AbAg,
\n",
    "            +ko2*Ab*AbAg-kd2*Ab2Ag,\n",
    "            -ko1*Ab*Ag+kd1*AbAg-ko2*Ab*AbAg+kd2*Ab2Ag,\n",
    "            -ko1*Ab*Ag+kd1*AbAg-ko3*Ag*AbAg]\n",
    "\n",
    "\n",
    "# Normalized agglutination versus antigen using two initial particle
concentrations\n",
    "def Agglu_2AbpConc(Args, \n",
    "                   AbConcs=[1,10], \n",
    "                   AgConcs=np.arange(0,40,step=1),\n",
    "                   Timepoint=1,\n",
    "                   TimeSpan=3,\n",
    "                   dpi=100):\n",
    "    \n",
    "    Lins=200\n",
    "    Tindex=int(Lins/TimeSpan*Timepoint)\n",
    "\n",
    "    #Figure: two particle concentrations\n",
    "    plt.figure(figsize=[4.5,3],dpi=dpi,layout=\"tight\")\n",
    "    plt.style.use('default')\n",
    "    i=0\n",
    "    Agglutination=[]\n",
    "    for AgConc in AgConcs:\n",
    "        sol = solve_ivp(Agglutination4_5, [0,TimeSpan],
[0,0,AbConcs[i],AgConc], args=Args,\n",
    "                         dense_output=True)\n",
    "        t = np.linspace(0, TimeSpan, Lins)\n",
    "        z = sol.sol(t)\n",
    "
#Agglutination.append((z.T[Tindex][1]+z.T[Tindex][0])/AbConcs[i])\n",
    "        Agglutination.append((z.T[Tindex][1])/AbConcs[i])\n",
    "        #print(AgConc)\n",
    "    plt.plot(AgConcs, Agglutination,linewidth=2)\n",
    "    plt.ylim(0,max(Agglutination)+0.2)\n",
    "\n",
    "    #plot the K parameters \n",
    "    FitParams = (r'$k_{on,1}$ = '+f'{Args[0]:5.2f}\\n'\n",
    "                 r'$k_{off,1}$ = '+f'{Args[1]:5.2f}\\n'\n",
    "                 r'$k_{on,2}$ = '+f'{Args[2]:5.2f}\\n'\n",
    "                 r'$k_{off,2}$ = '+f'{Args[3]:5.2f}\\n'\n",
    "                 r'$k_{on,3}$ = '+f'{Args[4]:5.2f}')\n",
    "    bbox = dict(boxstyle='round', fc='#F1FDED', ec='#2ca02c')\n",
```

```
    "    plt.text(max(AgConcs), max(Agglutination)+0.17, FitParams, 
fontsize='small', bbox=bbox, \n",
    "             ha='right', va=\"top\")\n",
    "\n",
    "    i=1\n",
    "    Agglutination=[]\n",
    "    for AgConc in AgConcs:\n",
    "        sol = solve_ivp(Agglutination4_5, [0,TimeSpan], 
[0,0,AbConcs[i],AgConc], args=Args,\n",
    "                        dense_output=True)\n",
    "        t = np.linspace(0, TimeSpan, Lins)\n",
    "        z = sol.sol(t)\n",
    "        
#Agglutination.append((z.T[Tindex][1]+z.T[Tindex][0])/AbConcs[i])\n",
    "        Agglutination.append((z.T[Tindex][1])/AbConcs[i])\n",
    "    plt.plot(AgConcs, Agglutination, linewidth=2)\n",
    "    \n",
    "    plt.xlabel('Antigen concentration')\n",
    "    plt.ylabel('Normalized agglutination')\n",
    "    plt.legend([str(AbConcs[0]), str(AbConcs[1])], loc=2,\n",
    "               title='Particle conc.\\n(relative value)', \n",
    "               title_fontsize='small',fontsize='small',\n",
    "               framealpha=0)\n",
    "    #plt.title('Agglutination Simulation')\n",
    "\n",
    "    plt.show()\n",
    "    plt.close()"
   ]
  },
  {
   "cell_type": "markdown",
   "id": "f8833cc8-ea55-4b2f-bc1e-c9f5b71143f1",
   "metadata": {
    "tags": []
   },
   "source": [
    "# **K Parameter Search**\n",
    "- 4 components\n",
    "- 5 parameters"
   ]
  },
  {
   "cell_type": "markdown",
   "id": "a3d2b51d-eaa4-4129-a1d6-59ceef425608",
   "metadata": {
    "tags": []
   },
   "source": [
    "## Parameter grid"
   ]
  },
  {
   "cell_type": "code",
   "execution_count": null,
```

```
   "id": "4dc9529a-b800-4c9d-89e3-36408793dba6",
   "metadata": {
    "scrolled": true,
    "tags": []
   },
   "outputs": [],
   "source": [
    "# Each appropriate combination of k will give a plot of normalized 
agglutination \n",
    "# simulated with two antibody-particle concentrations\n",
    "\n",
    "AbConcs=[5,10]\n",
    "AgConcs=np.arange(0,25,step=0.5)\n",
    "\n",
    "\n",
    "# Different grids for parameter search \n",
    "'''\n",
    "Param_Grids={'Kon1': np.arange(4,10,step=2),\n",
    "              'Koff1': np.arange(0.2,1,step=0.4),\n",
    "              'Kon2': np.arange(1,2,step=0.5),\n",
    "              'Koff2': np.arange(0.05,0.2, step=0.05),\n",
    "              'Kon3': np.arange(1,2,step=0.5)}\n",
    "\n",
    "'''\n",
    "Param_Grids={'Kon1': [2, 6, 10],\n",
    "              'Koff1': [0.1, 0.4, 1],\n",
    "              'Kon2': [0.5, 1],\n",
    "              'Koff2': [0.05, 0.5],\n",
    "              'Kon3': [1, 2]}\n",
    "\n",
    "\n",

"ParamCombine=pd.DataFrame(columns=['Kon1','Koff1','Kon2','Koff2','Kon3'])
\n",
    "for Kon1 in Param_Grids['Kon1']:\n",
    "    for Koff1 in Param_Grids['Koff1']:\n",
    "        for Kon2 in Param_Grids['Kon2']:\n",
    "            for Koff2 in Param_Grids['Koff2']:\n",
    "                for Kon3 in Param_Grids['Kon3']:\n",
    "                    if Kon1/Koff1>=Kon2/Koff2 and Kon1>Kon2 and 
Kon1>Kon3:\n",
    "                        Args = [Kon1, Koff1, Kon2, Koff2, Kon3]\n",
    "                        ParamCombine.loc[len(ParamCombine)] = 
Args\n",
    "                        Agglu_2AbpConc(Args, \n",
    "                                       AbConcs=AbConcs, \n",
    "                                       AgConcs=AgConcs)\n",
    "\n",
    "# show a table of paramters combination                        \n",
    "display(ParamCombine)"
   ]
  },
  {
   "cell_type": "markdown",
```

```
   "id": "63ccb66f-5191-425c-93c5-603cdcf72554",
   "metadata": {
    "tags": []
   },
   "source": [
    "## Figure: normalized agglutination"
   ]
  },
  {
   "cell_type": "code",
   "execution_count": null,
   "id": "9aad574f-4920-4aa6-a83d-114965f38fe6",
   "metadata": {
    "tags": []
   },
   "outputs": [],
   "source": [
    "# With one param combination, plot normalized agglutination and time
dynamics figure \n",
    "# High resolution using dpi=600\n",
    "\n",
    "Args=[6, 0.1, 1, 0.05, 2]\n",
    "Agglu_2AbpConc(Args, AbConcs=[5,10],
AgConcs=np.arange(0,25,step=0.1),\n",
    "               Timepoint=1,dpi=600)\n",
    "\n",
    "AbConc=10\n",
    "AgConc=10\n",
    "TimeSpan=3\n",
    "Timepoint=1\n",
    "Lins=200\n",
    "Tindex=int(Lins/TimeSpan*Timepoint) #the index of timepoint\n",
    "\n",
    "# Figures showing time-dependent dynamics of components in system\n",
    "plt.style.use('seaborn-v0_8-pastel') #seaborn-v0_8-pastel is the
light one\n",
    "plt.figure(figsize=[2.5,2.5],dpi=600,layout=\"tight\")\n",
    "sol = solve_ivp(Agglutination4_5, [0,TimeSpan], [0,0,AbConc,AgConc],
args=Args,\n",
    "                dense_output=True)\n",
    "t = np.linspace(0, 3, Lins)\n",
    "z = sol.sol(t)\n",
    "import matplotlib.pyplot as plt\n",
    "plt.plot(t,z.T, linewidth=2, linestyle='-')\n",
    "plt.xlim(0,1)\n",
    "plt.ylim(0,10)\n",
    "plt.xlabel('Time', fontsize='small')\n",
    "plt.ylabel('Relative conc.', fontsize='small')\n",
    "plt.legend(['Abp-Ag', 'Abp2-Ag', 'Abp',
'Ag'],fontsize='small',framealpha=0)\n",
    "#plt.title('Agglutination')\n",
    "plt.show()\n",
    "plt.close()"
   ]
```

```
  },
  {
   "cell_type": "markdown",
   "id": "a5ad4b9d-6199-40ee-abb8-678b669f2e0b",
   "metadata": {
    "tags": []
   },
   "source": [
    "## Figure: time-dependent dynamics"
   ]
  },
  {
   "cell_type": "code",
   "execution_count": null,
   "id": "77561788-9fea-409b-8d50-3d5b90e04764",
   "metadata": {
    "tags": []
   },
   "outputs": [],
   "source": [
    "# With selected antibody-particle concentrations and antigen
selections,\n",
    "# give example figures showing the time-dependent dynamics of each
component in system\n",
    "AbConcs=[5,10]\n",
    "AgConcs=[1, 5, 10]\n",
    "\n",
    "for AbConc in AbConcs:\n",
    "    for AgConc in AgConcs:\n",
    "        # Figures showing components over t\n",
    "        plt.rcParams.update(plt.rcParamsDefault)\n",
    "        plt.style.use('seaborn-v0_8-paper')\n",
    "
plt.figure(figsize=[3,2.5],dpi=600,layout=\"tight\",alpha=0)\n",
    "        \n",
    "        sol = solve_ivp(Agglutination4_5, [0,TimeSpan],
[0,0,AbConc,AgConc], args=Args,\n",
    "                        dense_output=True)\n",
    "        t = np.linspace(0, 3, Lins)\n",
    "        z = sol.sol(t)\n",
    "        plt.plot(t,z.T, linewidth=2, linestyle='-')\n",
    "        plt.xlim(0,2)\n",
    "        plt.ylim(0,max(AbConc,AgConc))\n",
    "        plt.xlabel('Time', fontsize='small')\n",
    "        plt.ylabel('Relative conc.', fontsize='small')\n",
    "\n",
    "        plt.legend(['Abp-Ag', 'Abp2-Ag', 'Abp (from
'+str(AbConc)+')', 'Ag (from '+str(AgConc)+')'],\n",
    "                   fontsize='small',framealpha=0)\n",
    "        #plt.title('Agglutination')\n",
    "        plt.show()\n",
    "        plt.close()"
   ]
  },
```

```
  {
   "cell_type": "markdown",
   "id": "26b498c2-c7d3-4a95-854b-683aba3cf07a",
   "metadata": {},
   "source": [
    "## Save the parameter combination as csv"
   ]
  },
  {
   "cell_type": "code",
   "execution_count": null,
   "id": "b339ae4b-d5c4-435e-b550-307ba310021d",
   "metadata": {
    "tags": []
   },
   "outputs": [],
   "source": [
    "ParamCombine.to_csv('ParamCombine.csv')"
   ]
  }
 ],
 "metadata": {
  "kernelspec": {
   "display_name": "Python 3 (ipykernel)",
   "language": "python",
   "name": "python3"
  },
  "language_info": {
   "codemirror_mode": {
    "name": "ipython",
    "version": 3
   },
   "file_extension": ".py",
   "mimetype": "text/x-python",
   "name": "python",
   "nbconvert_exporter": "python",
   "pygments_lexer": "ipython3",
   "version": "3.10.13"
  },
  "toc-autonumbering": false,
  "toc-showmarkdowntxt": false,
  "toc-showtags": false
 },
 "nbformat": 4,
 "nbformat_minor": 5
}
```
